# Hierarchically porous and mechanically stable monoliths from ordered mesoporous silica and their water filtration potential†

**DOI:** 10.1039/d2na00368f

**Published:** 2022-08-15

**Authors:** Laura M. Henning, Julian T. Müller, Glen J. Smales, Brian R. Pauw, Johannes Schmidt, Maged F. Bekheet, Aleksander Gurlo, Ulla Simon

**Affiliations:** Technische Universität Berlin, Faculty III Process Sciences, Institute of Material Science and Technology, Chair of Advanced Ceramic Materials Straße des 17. Juni 135 10623 Berlin Germany laura.m.henning@ceramics.tu-berlin.de +49 30 314 70483; Bundesanstalt für Materialforschung und -prüfung (BAM), Division 6.5 – Polymers in Life Sciences and Nanotechnology Unter den Eichen 87 12205 Berlin Germany glen-jacob.smales@bam.de +49 30 8104 3314; Technische Universität Berlin, Faculty II Mathematics and Natural Sciences, Institute of Chemistry, Chair of Functional Materials Straße des 17. Juni 135 10623 Berlin Germany

## Abstract

Mechanically stable structures with interconnected hierarchical porosity combine the benefits of both small and large pores, such as high surface area, pore volume, and good mass transport capabilities. Hence, lightweight micro-/meso-/macroporous monoliths are prepared from ordered mesoporous silica COK-12 by means of spark plasma sintering (SPS, S-sintering) and compared to conventionally (C-) sintered monoliths. A multi-scale model is developed to fit the small angle X-ray scattering data and obtain information on the hexagonal lattice parameters, pore sizes from the macro to the micro range, as well as the dimensions of the silica population. For both sintering techniques, the overall mesoporosity, hexagonal pore ordering, and amorphous character are preserved. The monoliths' porosity (77–49%), mesopore size (6.2–5.2 nm), pore volume (0.50–0.22 g cm^−3^), and specific surface area (451–180 m^2^ g^−1^) decrease with increasing processing temperature and pressure. While the difference in porosity is enhanced, the structural parameters between the C-and S-sintered monoliths are largely converging at 900 °C, except for the mesopore size and lattice parameter, whose dimensions are more extensively preserved in the S-sintered monoliths, however, coming along with larger deviations from the theoretical lattice. Their higher mechanical properties (biaxial strength up to 49 MPa, 724 MPa HV 9.807 N) at comparable porosities and ability to withstand ultrasonic treatment and dead-end filtration up to 7 bar allow S-sintered monoliths to reach a high permeance (2634 L m^−2^ h^−1^ bar^−1^), permeability (1.25 × 10^−14^ m^2^), and ability to reduce the chemical oxygen demand by 90% during filtration of a surfactant-stabilized oil in water emulsion, while indicating reasonable resistance towards fouling.

## Abstract 2

The sintering behavior of OMS COK-12 into mechanically stable, porous monoliths was analyzed, including a customized multi-scale SAXS model.

## Graphical abstract

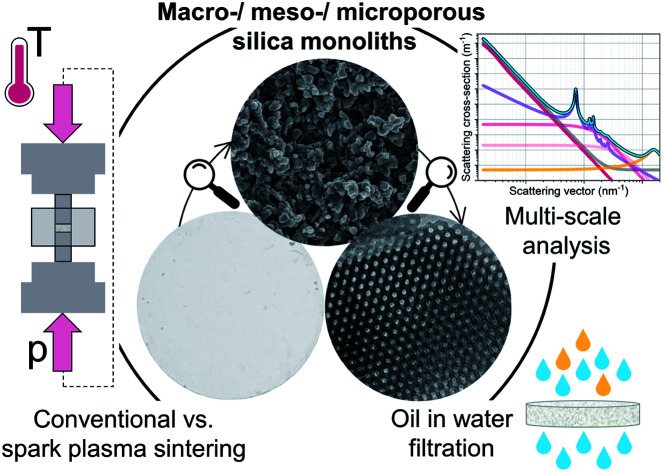

## 1 Introduction

Ordered mesoporous silica (OMS) materials are commonly used as nano-sized powders, which are usually obtained directly from synthesis.^1,2^ While OMS nanoparticles (NPs) are suitable for many applications such as (surface functionalized) adsorbents and supports for (photo)catalytic and drug delivery systems,^3–6^ several drawbacks constrain their application. Firstly, NP handling requires extensive safety precautions to avoid dust inhalation and associated hazards, hampering large-scale industrial usage. Secondly, NP powder beds usually exhibit high pressure drops during operation, while NP suspension systems normally require high energy or long times for NP segregation. Lastly, the ecofriendly and efficient recovery of NPs for reutilization remains a challenge.

The coating of mesoporous films on various substrates is developed as one pathway to overcome those challenges. Therefore, OMS precursors are usually deposited by dip coating or spin coating onto a membrane substrate.^7^ Alternatively, OMS monoliths are directly obtained by sol–gel processes, spinodal decomposition, solvothermal routes, hard/soft/ice templating, frothing, as well as combinations of those techniques.^8–14^ However, little or no information is available about their mechanical properties. Their low processing temperatures suggest overall weak mechanical resistances, which might hinder deployment in pressure-loaded applications such as filtration. Additionally, a great number of listed applications would benefit from hierarchical pore structures, allowing to combine high specific surfaces and pore volumes with high mechanical properties while benefitting from high accessibility and controlled mass transfer.

Spark plasma sintering (SPS), also known as field-assisted sintering technique (FAST), pulsed electric current sintering (PECS), electric discharge sintering (EDS), or pulsed current processing (PCP), is a promising one-step technique for rapid powder consolidation to obtain mechanically robust single-phase OMS monoliths. During SPS, uniaxial pressure is applied on the powder while an electric current is deployed on the electrically conductive pressing die, usually made from graphite, to facilitate sintering by Joule heating in a controlled atmosphere, although neither sparks nor plasma have been observed yet.^15,16^ In comparison to conventional sintering techniques, high heating and cooling rates, along with short dwell times in the range of seconds or minutes, allow for rapid, one-step processing with the possibility for grain growth adjustment, while no pre-compaction of the powder is required.^17^ Initial restrictions of the SPS process, such as scalability or structural limitations, have been overcome by the development of multi-sample dies, machinable parts, and semi-continuous systems.^18,19^ While one focus of SPS remains on the densification of conventional and hard-to-sinter materials, recently, the production of porous products came into focus.^20,21^ SPS was previously applied to fabricate porous OMS monoliths from sol–gel derived SBA-15 powder,^22,23^ mesoporous spherical particles,^24^ and hydrothermally produced OMS.^25^ The studies used the nonionic block copolymer Pluronic P123 as a templating agent, the latter one also Pluronic L121 and F127, resulting in ordered structures of hexagonal, lamellar, and cubic character, respectively. However, current literature both lacks and demands a comparative analysis of porous materials prepared by different sintering techniques, but with similar pore fraction and size.^21^ Furthermore, the influence of the SPS conditions on the mechanical properties of porous sintered materials remains marginally researched.

The aim of this work is to produce and thoroughly characterize binder-free, hierarchically porous monoliths from one large batch of more environmentally friendly synthesized OMS COK-12 powder^8,26,27^ by solid state sintering methods, namely SPS as well as conventional sintering, to enhance the understanding of OMS sintering and the effect of sintering pressure and temperature on the micro-, meso-, and macroporosity, among other properties. Therefore, an elaborate, custom small angle X-ray scattering (SAXS) model for powder and partially sintered hexagonal OMS is presented. This obtains a thorough structural examination further complemented by additional characterization techniques. Additionally, because of the structural differences, the monolith's mechanical properties, their water permeability and suitability for separation applications, and COK-12's higher sintering resistance compared to SBA-15 are discussed.

## 2 Experimental

### 2.1 Chemicals

Pluronic P123 (*M*_W_ ∼ 5800 g mol^−1^) and Tween 80 were obtained from Sigma-Aldrich (Merck, Germany). Citric acid (≥99.5%, anhydrous), trisodium citrate dihydrate (≥99%), and sodium silicate (7.8–8.5 wt% Na_2_O, 25.8–28.5 wt% SiO_2_) were purchased from Carl Roth (Germany). Hydraulic oil Tellus Oil 46 with a kinematic viscosity of 46 mm^2^ s^−1^ (40 °C) was acquired from Shell (Shell Deutschland Oil GmbH, Germany). Deionized water (DIW) was used for the synthesis and testing.

### 2.2 COK-12 synthesis

The synthesis of COK-12 was performed as previously reported.^8,26,28^ A batch upscaled by the factor of 50 was produced as follows. Firstly, 200 g P123 was dissolved in 5375 mL DIW. After P123 dissolution, 168.1 g anhydrous citric acid and 144.1 g trisodium citrate dihydrate were added to buffer the solution. After stirring for 24 h, a solution of 520 g sodium silicate and 1500 mL DIW was incorporated into the buffered solution. An immediate solid formation was observed, and stirring was maintained for 5 min, after which the slurry was aged for 24 h without stirring. Subsequently, the solid was separated from the synthesis solution by vacuum filtration and washed with 25 L DIW. Finally, the material was dried at 60 °C overnight and calcined in air at 500 °C with a 1 K min^−1^ heating ramp and 8 h dwell time to remove the templating agent.

### 2.3 Spark plasma sintering of COK-12

Spark plasma sintering, subsequently referred to as S-sintering, of COK-12 was performed in custom, cylindrical three-sample graphite dies of 3× ∅10 mm in a parallel configuration, see Fig. S1,† using a Desktop Sinter System SPS-211Lx (Dr Sinter Lab Jr., Japan) under vacuum. The weighed portion, heating rate, and dwell time were constant with 0.11 g COK-12 per sample, 100 K min^−1^, and 1 min, respectively. Pressures of 2.5 MPa, 12.5 MPa, 25 MPa, and 50 MPa and temperatures of 600 °C, 700 °C, 800 °C, and 900 °C were applied. For reference, dense samples were produced at 50 MPa and 1045 °C using 0.22 g COK-12 per sample, a heating rate of 25 K min^−1^, and a dwell time of 45 min.

### 2.4 Conventional sintering of COK-12

For comparison, conventional sintering, subsequently referred to as C-sintering, was performed by uniaxial pressing of COK-12 in a universal testing machine Z020 (ZwickRoell, Germany) at 25 MPa and 50 MPa. Equally, the weighed portion was 0.11 g COK-12 per sample. After pressing, the samples were subsequently sintered at 600 °C, 700 °C, 800 °C, and 900 °C with a dwell time of 12 h in a muffle furnace (Nabertherm, Germany).

### 2.5 Characterization

The apparent porosity and bulk density of the monoliths were determined using Archimedes' method based on ISO 18754:2020.^29^ Impregnation was carried out by the boiling method.

The biaxial strength was ascertained using the ball-on-three balls (B3B) test.^30^ In comparison to the common ring-on-ring test, the B3B test minimizes fraction and inhomogeneous load distribution and is suitable for investigating small, as-processed disc-shaped samples.^31^ Three as-processed silica monoliths per parameter set were tested in a universal testing machine Z020 (ZwickRoell, Germany) equipped with a 20 kN load cell using high precision bearing balls of 6.5 mm in diameter. Testing was performed with a loading rate of 1 N s^−1^. The biaxial strength was calculated using a Poisson's ratio of 0.3 for mesoporous silica.^32^ A Weibull analysis was performed on a set of 30 S-sintered monoliths processed at 800 °C and 12.5 MPa using the default Weibull probability plot function in Origin 2021b (OriginLab Corporation, USA).

The Vickers hardness was ascertained on a Z3212 hardness tester (ZwickRoell, Germany) based on EN ISO 14705:2021 with a test force of 9.807 N applied for 15 s.^33^ Therefore, the samples were embedded in Epofix epoxy resin (Struers, Germany), ground, and polished to 3 μm. Indents from the central part of the polished cross sections were measured using a DM 4000 M light microscope (Leica, Germany).

The biaxial strength and Vickers hardness were fitted using the Ryshkewitch model, yielded by Duckworth^34^1
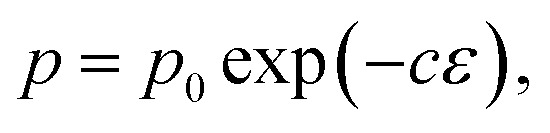
the Hashin model^35^2
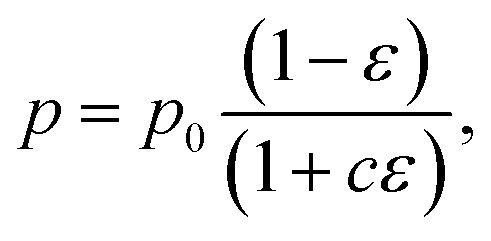
and Bal'shin model^36^3
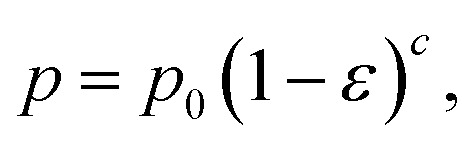
where *p* is the property of the material with the porosity *ε*, *p*_0_ is the property at zero porosity, and *c* is the respective fitting parameter. Furthermore, the mechanical properties were fitted using the percolation law4
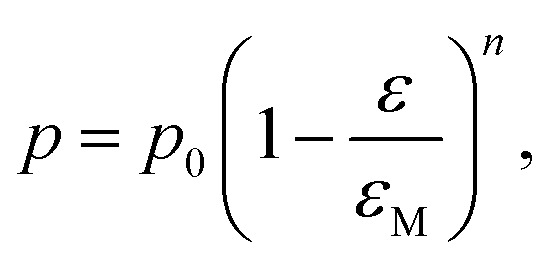
where *ε*_M_ is the maximum porosity of the powder mass, and *n* is the fitting parameter.^37^*ε*_M_ was ascertained to be 0.95 by using the COK-12 powder apparent density of 0.1 g cm^−3^ and COK-12 true density of 2.2 g cm^−3^.

The pore structure, pore size, pore volume, and specific surface area of the COK-12 powder and monoliths were studied using nitrogen sorption analysis in a QuadraSorb apparatus (Quantachrome, USA). Isotherms were recorded at 77 K after degassing under vacuum for 10 h at 200 °C. The surface area was determined using the Brunauer, Emmett and Teller (BET) method. Model applicability was ensured using the Rouquerol plot.^38,39^ The mesopore sizes were estimated based on non-local density functional theory (NLDFT) calculations using the adsorption branch of the isotherm and a cylindrical geometry. All nitrogen sorption data was analyzed using the QuadraWin software (Quantachrome, USA). Mercury intrusion porosimetry was conducted on a Porosimeter 2000 (Carlo Erba Instruments, Italy) to extend the measuring range to macropores for S-sintered monoliths processed at 800 °C and 12.5 MPa. The classification of the pore sizes within this work was undertaken according to the IUPAC definition.^39^

Small-/wide-angle X-ray scattering (SAXS/WAXS) measurements were performed on the heavily customized MOUSE instrument at the Bundesanstalt für Materialforschung und -prüfung (BAM).^40^ Here, monochromatized Cu Kα (*λ* = 1.5406 Å) X-rays were generated from a sealed-tube micro-source, and data was collected on an in-vacuum Eiger 1 M detector (Dectris, Switzerland), which was placed at multiple distances between 57 and 2507 mm from the sample. The monoliths, cut into 1 mm slices using a Model 3400 diamond wire saw (Well Diamantdrahtsägen GmbH, Germany), and the loosely compacted COK-12 powder were measured between two pieces of Scotch Magic™ tape. The obtained data was then processed to an absolute intensity scale using the DAWN software package according to extensive (standardized) data correction procedures, which include a thorough assessment of the measurement uncertainties.^41,42^ Fitting of the SAXS data was performed using SASfit,^43^ allowing to obtain information on the lattice parameter, macropore size, mesopore size, two populations of micropores, as well as the silica peak position and width. More detailed information on the SAXS modelling can be found in the ESI (Fig. S2–S8†).

The wall thickness *w*_t_ and wall area *w*_a_ were calculated according to

5 *w*_t_ = *a*_0_ − *D*,

and6
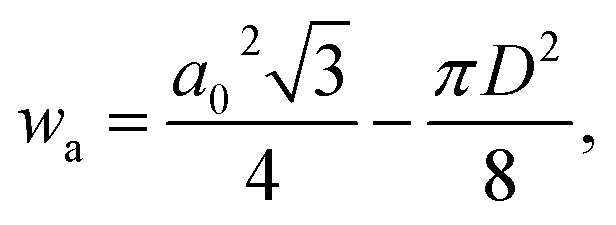
respectively, where *a*_0_ and *D* are the lattice parameter and pore diameter, respectively, as obtained by SAXS.^44,45^

X-ray total scattering data of S-sintered COK-12 monoliths processed at 25 MPa, ground to powder, was collected at beamline P02.1, PETRA III at the DESY at a wavelength of 0.20735 Å at a sample to detector distance of 280 mm using a Varex XRD 4343CT detector. A LaB_6_ (NIST 660c) standard and an empty capillary were measured to account for instrumental contributions and capillary glass contribution, respectively. 2D data was processed using Dioptas 0.5.2.^46^ Pair distribution functions (PDFs) were processed from the total scattering data using PDFgetX3 with *Q*_max_ = 16 Å^−1^ and *Q*_maxinst_ = 24 Å^−1^.^47^

X-ray diffraction (XRD) patterns were collected on COK-12 powder and monoliths produced at the highest processing pressure of 50 MPa using a Bruker D8 ADVANCE with Cu Kα radiation (*λ* = 1.5406 Å), operated at 40 kV in Bragg–Brentano geometry with a LINXEYE 1D detector (Bruker, Germany).

Visualization of the surface morphology, including sintering necks, was performed on the fractured surfaces after B3B testing by means of scanning electron microscopy (SEM) using a LEO Gemini 1530 (Zeiss, Germany) at 5 kV with an InLens electron detector and an aperture size of 30 μm after sputtering with a thin carbon layer.

The mesostructure of an S-sintered COK-12 monolith processed at the highest pressure and temperature, *i.e.*, 50 MPa and 900 °C, respectively, was visualized with a transmission electron microscope (TEM) in a FEI Tecnai G^2^ 20 S-TWIN (FEI, USA) equipped with a LaB_6_-source at 200 keV acceleration voltage upon grinding. Images were recorded with a GATAN MS794 P CCD-camera.

Pure water permeability was determined using a custom-built dead-end filtration setup as previously reported.^48,49^ Therefore, the samples were cleaned in DIW for 10 s using a Sonorex RK 52 H (Bandelin, Germany) ultrasonic bath. Following, the samples were inserted into the stainless steel container and fixed with O-rings, resulting in an available sample area of 28.3 mm^2^. Subsequently, the container was filled with DIW and pressurized with synthetic air up to pressures of 1, 2.5, 4, 5.5, and 7 bar, respectively. The mass of the permeated DIW was automatically recorded every 5 s using an Adventurer Analytical precision scale (OHAUS, USA) until either 75 g DIW were collected or 30 min passed, whichever occurred first. Afterwards, the volumetric flux at each pressure was calculated according to7
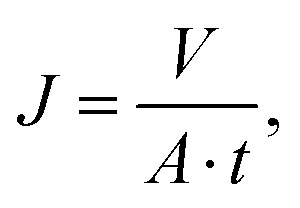
where *J* is the flux in L m^−2^ h^−1^, *V* is the volume of permeated water in L, *A* is the sample area in m^2^, and *t* is the permeation time in h. The water permeance *L*_p_ in L m^−2^ h^−1^ bar^−1^ was obtained from the slope of the linear regression of the flux–pressure curve according to the linear relationship

8 *J* = *L*_p_Δ*p*,

where Δ*p* is the pressure drop in bar. The permeability *k* in m^2^ was calculated according to Darcy's law for single phase flow according to

9 *k* = *L*_p_*ηl*,

using the water permeance in m^3^ m^−2^ s^−1^ Pa^−1^, the dynamic viscosity of water *η* in Pa s, and the thickness *l* of the membrane in m. Permeability data was fitted using the Kozeny–Carman equation10
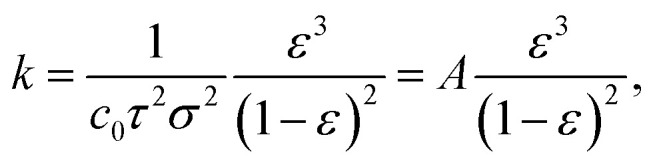
where *c*_0_ is the Kozeny coefficient, *τ* is the tortuosity, *σ* is the specific surface area with respect to the unit volume of the solid matrix in m^2^ m^−3^, *ε* is the unitless porosity and *A* is the Cozeny–Karman parameter.^50–52^

For the filtration experiment, a surfactant-stabilized oil in water emulsion with an oil concentration of 100 mg L^−1^ was prepared by mixing 0.2 g hydraulic oil with 25 mL of 2 g L^−1^ Tween 80 in DIW and made up to 2 L with DIW and blending with a hand blender (Kult S; WMF, Germany) for 1 min at medium speed. Afterwards, the emulsion was degassed in a vacuum cabinet. The initial oil droplet size distribution was ascertained using a laser particle size analyzer LS 13 320 (Beckman Coulter, USA). Oil in water filtration was performed in the same custom dead-end filtration setup described above at 1 bar on an S-sintered COK-12 monolith processed at a pressure of 12.5 MPa and a temperature of 800 °C. Filtration was performed for 40 min. The oil restraint was assessed by measuring the chemical oxygen demand (COD) before and after filtration by dichromate oxidation in a DR 5000 spectrophotometer (Hach Lange GmbH, Germany).

## 3 Results and discussion

The results of the nitrogen sorption analysis, shown in Fig. S9,† reveal type IVa isotherms with type H1 hysteresis loops for COK-12 powder and S- and C-sintered COK-12 monoliths, characteristic for mesoporous materials with a narrow pore size distribution such as COK-12.^39^ With increasing pressure and temperature, the hysteresis loops decrease in size and are shifted towards lower relative pressures (earlier for S- than C-), indicating a decrease in pore size and pore volume. Furthermore, the slope of the hysteresis gradually decreases with increasing pressure and temperature, more particularly, which is more pronounced in the S- than in the C-sintered monoliths, indicating irregularities in the pore diameter. Thereby, the adsorption and desorption branches of the H1 hysteresis loop remain approximately parallel, but slightly convex, indicating a minor change in pore geometry, yet no significant pore blocking or the presence of complex structures. Interestingly, at pressures above *ca.* 0.85 *p*/*p*_0_ a second hysteresis loop can be found, which is most pronounced for S-sintered monoliths produced at the highest temperature and pressure. Due to the limited amount of measurement points in this area, an assessment of the hysteresis loops' slope is delicate. However, the presence of a second hysteresis loop at higher relative pressures implies the presence of macropores larger than *ca.* 50 nm. Those can be associated to interparticle pores, resulting from the cavity compaction between the COK-12 particles, and are in line with what has been reported previously.^23^

In accordance, the NLDFT pore size distributions reveal a decrease in modal mesopore diameter and pore volume with increasing pressure and temperature, as shown in Fig. S10.† Micro- and large macropore analysis are omitted, as they were not measured or cannot be adequately estimated by NLDFT. However, the micropore development can be appraised by the truncated peak around 2 nm, revealing a significant loss in microporosity for all sintered monoliths. Within the mesopore range, the modal pore diameter of the S-sintered monoliths initially rises to 6.6 nm and then remains at 6.1 nm, corresponding to the mesopore size of the COK-12 powder, compare Fig. S11.† Subsequently, the modal pore diameter of the S-sintered monoliths produced at 700 °C and 800 °C at the lowest (2.5 MPa) and highest (50 MPa) pressures declines to 5.9 nm, and finally to 5.7 nm at 900 °C, regardless of the pressure. For the C-sintered COK-12 monoliths, a linear decrease in the pore diameter down to 5.3 nm at 900 °C is apparent. It is noticeable that the computation of the modal pore size by NLDFT yields only distinct pore sizes, allowing for only limited meaningfulness with data points so close. This inaccuracy in the pore size analysis can be ascribed to the small steps in the calculated theoretical isotherms, which do not account for chemically and geometrically heterogeneous surfaces.^39,53^

**Fig. 1:**
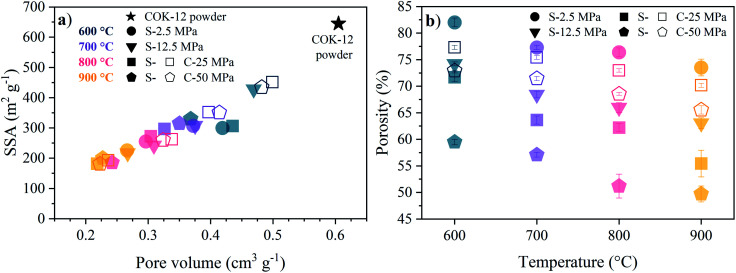
Sintering temperature and pressure dependence of (a) the BET specific surface area (SSA) and pore volume (NLDFT, adsorption branch) and (b) the apparent porosity as obtained from Archimedes' method for COK-12 powder as well as S- and C-sintered COK-12 monoliths.

The decrease in the mesopore size is associated with a loss in pore volume and a decrease in specific surface area and apparent porosity, as depicted in Fig. 1. Thereby, the linear decrease indicates a reduction of the pore sizes in the monoliths. The sintering temperature has a more pronounced influence on the BET specific surface area (SSA) and pore volume in comparison to the effect of the applied pressure, as can be seen from Fig. 1a. The higher preservation of these structural properties in C-sintered monoliths over S-sintered monoliths is greatly reduced with increasing temperature, resulting in a vanishing low difference between S- and C-sintered monoliths at 900 °C. In detail, starting from 645 m^2^ g^−1^ BET SSA for the original COK-12 powder, 69% and 49% of the BET SSA were preserved at 600 °C and higher pressures for C- and S-sintered monoliths, respectively, before declining to *ca.* 29% at 900 °C, compare Table S1.† Similarly, the initial pore volume of 0.61 cm^3^ g^−1^ reduces to 80% and 66% at 600 °C for the C- and S-sintered monoliths, respectively, and finally decreases to *ca.* 37% at 900 °C. It should be noticed that the pore volume obtained by NLDFT does not reflect the total monolith's pore volume, but only the pore volume up to macropores of *ca.* 80 nm. Hence, the apparent porosity, including the total macroporosity as determined by the Archimedes method, is displayed in Fig. 1b. A decreasing porosity with increasing temperature and pressure can be observed for both the C- and S-sintered monoliths, whereas the reduction in porosity is more distinct for the S-sintered monoliths in comparison to the C-sintered monoliths due to the combined application of pressure and temperature resulting in enhanced densification. The overall porosities are high, reaching up to 82% for the lowest pressure and temperature set (2.5 MPa, 600 °C) and preserving 50% for the highest pressure and temperature parameter set (50 MPa, 900 °C), even for the S-sintered monoliths. Thereby, for all monoliths, closed porosity is negligibly low as the apparent solid density remains approximately constant for all monoliths, as shown in Table S1.†

**Fig. 2:**
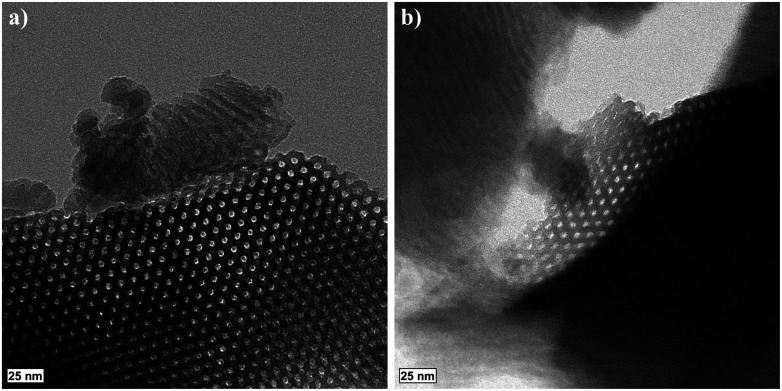
TEM images of a ground S-sintered COK-12 monolith processed at 50 MPa and 900 °C show the significant preservation of the hexagonally ordered mesopores (a and b) and areas of pore deformation (b).

The TEM images of a ground, S-sintered monolith, processed at the highest pressure and temperature, *i.e.*, 50 MPa and 900 °C, respectively, displayed in Fig. 2, show the distinct preservation of the hexagonally ordered mesopores within the COK-12 and thus, confirm the nitrogen sorption results. In some areas, such as in the upper center of Fig. 2b, pore deformations towards slit-like pores can be observed, which were proven credible upon sample reorientation. TEM images of unprocessed COK-12 powder for comparison are available in the literature.^28,45^

**Fig. 3:**
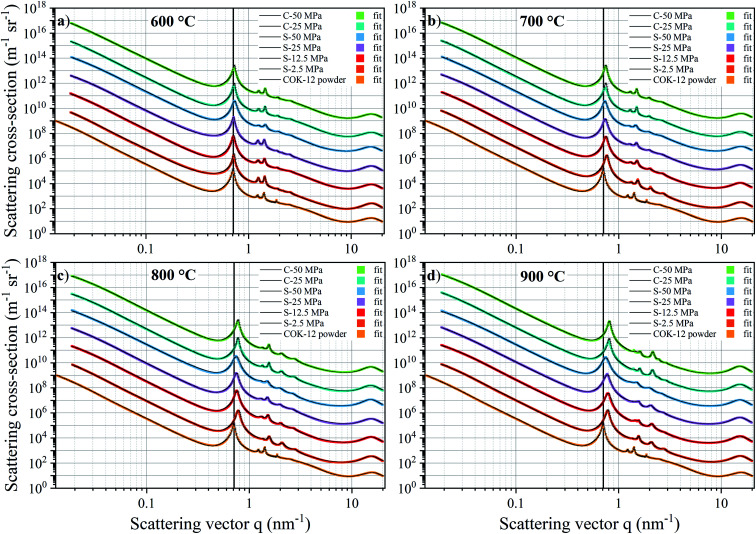
SAXS patterns and corresponding fits for the COK-12 powder and monoliths produced by S- and C-sintering with y-offsets at (a) 600 °C, (b) 700 °C, (c) 800 °C, and (d) 900 °C at varying pressures. Vertical lines are added to aid the eye in observing the peak shift.

**Fig. 4:**
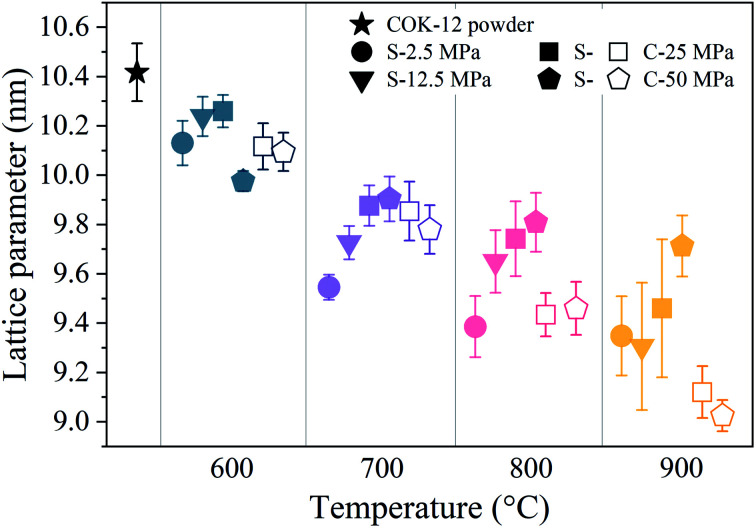
Lattice parameters of the hexagonal structure of the COK-12 powder and S- and C-sintered monoliths. Within the temperature columns, the data points are horizontally spaced for legibility. The error bars show the mean deviation between the expected theoretical lattice peak positions for the (11), (20), and (21) reflections included in the analysis, hinting towards the presence of strain or disorder.

The SAXS data and the corresponding fits are shown in Fig. 3. All patterns exhibit at least four well-resolved diffraction reflections at distances of *q*_1_ : *q*_2_ : *q*_3_ : *q*_4_ = 1 : 
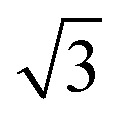
 : 
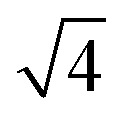
 : 
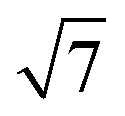
, satisfying the conditions for the 2D hexagonal symmetry *P*6*m* and thus, can be indexed as (10), (11), (20), and (21). With increasing sintering temperature, the reflections considerably shift towards higher scattering vectors, indicating a decrease in *d*-spacing, and, accordingly, a decrease in lattice parameter, as depicted in Fig. 4. Starting with a lattice parameter of 10.4 nm for the COK-12 powder, the hexagonal unit of the monoliths is contracted by up to *ca.* 13% when processed by C-sintering at the highest pressure and temperature, as listed in Table S1.† A roughly linear decrease of the lattice parameter can be observed with temperature for C-sintered monoliths, merely influenced by the processing pressure. In comparison, the decrease in the lattice parameter is less pronounced at higher pressures, in particular at higher temperatures, for S-sintered monoliths. Due to their geometrical linkage, the overall behavior of the lattice parameter with processing pressure and temperature is similar to the development of the mesopore sizes discussed previously. It can be supposed that at higher pressures, despite the thermal energy supplied, atom movement is more restricted and hence, equilibrium is not reached within the short duration of the S-sintering process, resulting in a higher preservation of the unit cell size. However, with higher temperatures, the overall deviation of the lattice parameter from the theoretical hexagonal lattice, represented in the form of error bars in Fig. 4, increases markedly for the S-sintered monoliths, while the influence of pressure is only minor. In contrast, the deviation from the theoretical hexagonal lattice remains approximately constant for the C-sintered monoliths for both varying temperatures and pressures. It can be derived that due to the simultaneous application of pressure and temperature during the S-sintering, temperature-induced stresses are intensified, which during C-sintering can be compensated by unrestricted readjustment of the sample diameter and height.

**Fig. 5:**
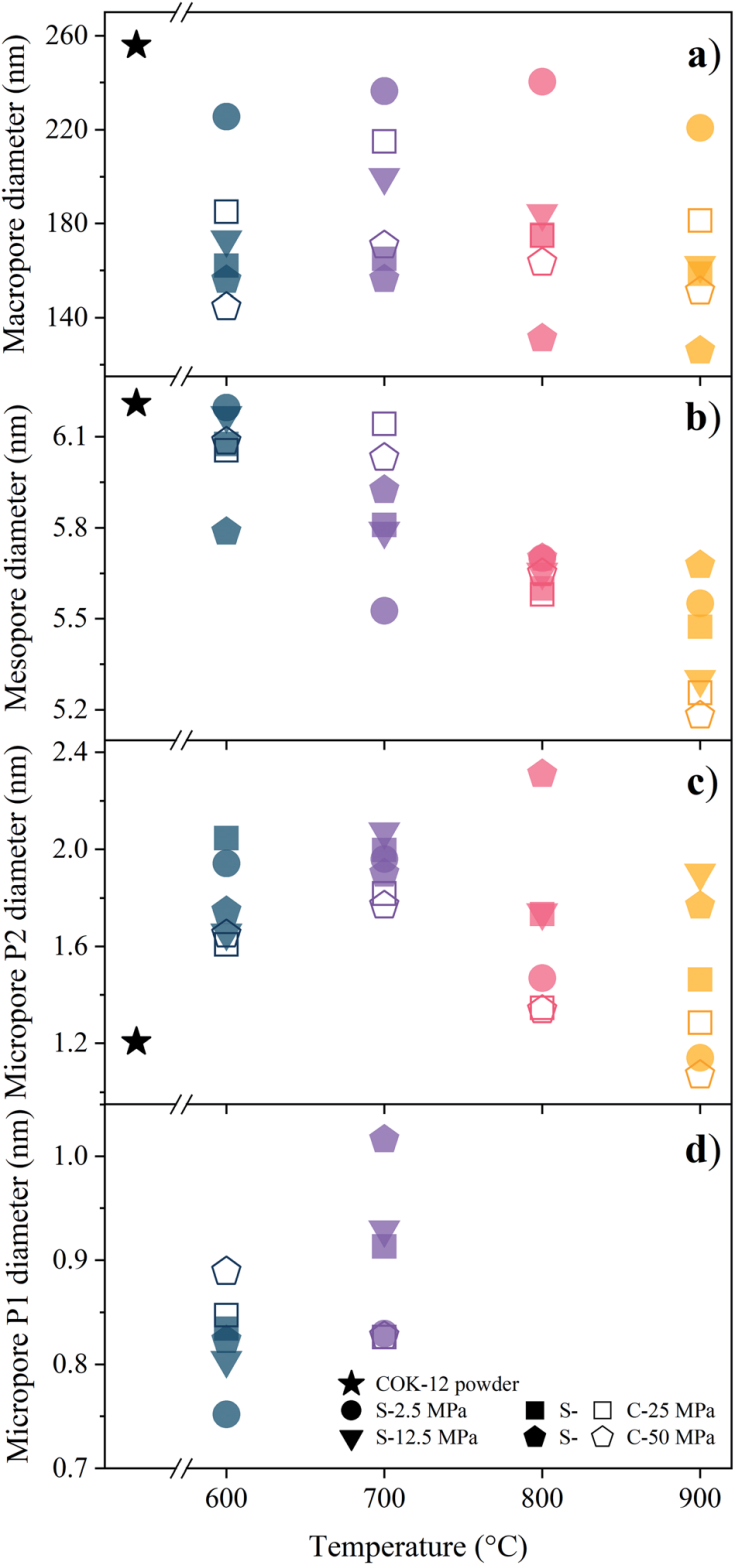
(a) Macro-, (b) meso-, and (c, d) micropore (P2, P1) diameters obtained from fitting the SAXS data of COK-12 powder and S- and C-sintered monoliths. No P1 micropores were determined for the COK-12 powder and the monoliths sintered at 800 and 900 °C.

As described, the fitting of the SAXS data additionally allows for pore size determination from the micro- to the macroscale scale, *i.e.*, from *ca.* 1–350 nm, resulting from the q-range limits of the SAXS measurements. However, larger pores are expected to also be present, with their size estimated by means of SEM and mercury intrusion porosimetry, as discussed below. The mean pore sizes derived from the SAXS fittings are displayed in Fig. 5. While the mesopores are homogeneous in size due to the assumed polydispersity of 10%, compare the exemplary data for the COK-12 powder in Fig. S12,† a substantial polydispersity can be found for the macro- and micropores, whose corresponding log-normal volume-weighted pore size distributions are shown in Fig. S13–S15.†

The mean macropore diameter, initially being 256 nm for the loosely compacted COK-12 powder, is only slightly reduced at 2.5 MPa, before decreasing below 220 nm with increasing temperature and pressure for both S- and C-sintered monoliths, as shown in Fig. 5a. However, the mean macropore diameter of the C-sintered monoliths is up to 50 nm larger than for the S-sintered monoliths, which can be attributed to the absence of pressure during the sintering step in the conventional sintering process, eliciting less densification. In contrast to the evolution of the lattice parameter, the diminution in the main macropore size is enhanced at high temperatures, in particular at high pressures, which can be attributed to the progressive elimination of interparticle voids. Hence, the volume-weighted macropore size distributions in Fig. S13† show a distinct increase in the volume of these macropores for C-sintered monoliths and S-sintered monoliths at lower pressures, presumably resulting from the compaction of larger macropores into the SAXS measurement range.

The general trend of decreasing pore size with increasing temperature and pressure is also apparent for the mean mesopore diameters shown in Fig. 5b. While the mesopore diameter remains approximately constant at *ca.* 6.1 nm for monoliths processed at 600 °C for both sintering techniques, a linear decrease of the mesopore diameter, down to 5.2 nm, with increasing temperature can be observed for C-sintered monoliths, with only a slight influence from the processing pressure. No such clear trend can be observed for the S-sintered monoliths. A comparison of the mesopore sizes obtained from SAXS and nitrogen sorption/NLDFT, see Table S1,† reveals a high accordance between the methods' results, exhibiting an average and maximum deviation of 0.2 and 0.5 nm, respectively. Considering the limited set of default diameters that can be obtained from NLDFT analyses, the presented SAXS fitting can be considered a more robust method for obtaining mesopore diameters. The mechanism of mesopore reduction with heat treatment for SBA-15, structurally comparable to COK-12, is reported to be due to contraction of the cylindrical pores without considerable changes in the length of the pore channels and elimination of intrawall porosity and has previously been observed for SBA-15 and COK-12.^28,54,55^ As the rigid, plate-like particles do not allow for any significant reduction of the pore channel lengths, barely any additional contraction is expected to occur even under higher pressures.

In addition to the macro- and mesopores, two populations of micropores could be identified, *i.e.*, smaller ones in the range of 0.75–1.02 nm and larger ones in the range of 1.2–2.31 nm, referred to as micropores of the population 1 (P1) and 2 (P2), respectively, as shown in Fig. 5c and d. P2 micropores are likely to be associated to the pores, which have previously been generated in the silica walls upon removal of the hydrophilic polyethylene oxide tails of the triblock copolymer.^28,56–58^ With increasing temperature and pressure, initially an increase in the P2 micropore size can be observed from 1.21 nm for the COK-12 powder up to 2.31 nm for S-sintered monoliths at 800 °C and 50 MPa, as depicted in Fig. 5c. While increasing micropore P2 diameters can be observed for S-sintered monoliths at high pressures, possibly due to crack growth, micropore consolidation, or distinct mesopore blocking, above 700 °C a decrease can be seen for low-pressure-sintered and C-sintered monoliths, possibly due to enhanced mesopore blocking or surface-smoothing silanol condensation in the micropores at high temperatures, resulting in the elimination of intrawall microporosity.^54,55^ This view is encouraged by the decrease in micropore volume with temperature, as shown in the volume-weighted pore size distributions of the P2 micropores in Fig. S14.† Furthermore, the development of the P2 micropore sizes might be associated to the random orientation of the particles, which result in a micropore crushing or micropore expansion for micropores aligned perpendicular or along the axis of applied pressure, respectively. Thereby, the loss of pore volume due to collapsed micropores cannot be compensated by the additional volume gained by micropore expansion, which can be seen from the decreased micropore contribution to the SAXS scattering patterns and nitrogen sorption data in comparison to the COK-12 powder, compare Fig. 3 and S10.† Overall, the decrease in P2 micropore size is less pronounced for the S-sintered monoliths, which can be associated to the shorter processing times. Additionally, P1 micropores could be found for the monoliths processed at 600 and 700 °C, shown in Fig. 5d, yet not for those processed at 800 and 900 °C, independent of the sintering technique, as well as for the COK-12 powder. Hence, the P1 micropores might directly be related to the processing and could be associated to intraparticle cracks, which emerged upon processing, yet vanished again due to enhanced micropore collapse at higher temperatures. Accordingly, a decrease in the micropore volume with increasing temperature can be observed in the volume-weighted pore size distributions of the P1 micropores in Fig. S15.† In contrast, inconsistent results on micropore information were obtained from the *I*_11_/*I*_20_ intensity ratio of the SAXS patterns in the literature,^54^ which can be ascribed to the neglected interplay between the hexagonal structure factor and cylindrical form factor, which was accounted for in the present model, as can be seen from Fig. S3 and S6† and the corresponding sections in the ESI.† These results highlight the importance of collecting a wide q-range for such hierarchical systems, so that information on the interplay of different pore structures can be elucidated and exploited. SAXS also has the benefit of being able to access closed pores contained within a system, due to differences observed in the contrast.^59^ With wide-ranging SAXS data it is possible to ascertain information on both micro- and macro-structures within a sample, even when such structures are only apparent in small volumes, compare the micropore size distributions obtained by nitrogen sorption depicted in Fig. S16.†

**Fig. 6:**
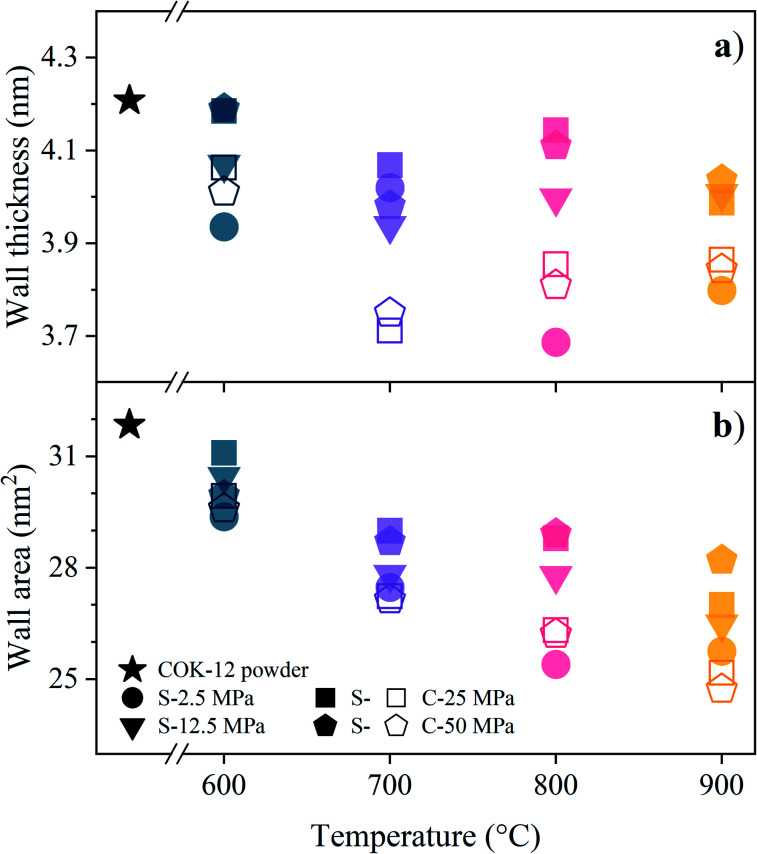
(a) Wall thickness and (b) wall area calculated according to eqn (5) and (6), respectively, from the SAXS data for COK-12 powder and S- and C-sintered monoliths.

The influence of the processing conditions on the parameters wall thickness and wall area, calculated according to eqn (5) and (6), respectively, derived from the SAXS lattice parameters and mesopore sizes, is shown in Fig. 6. The powder wall thickness of 4.2 nm is maintained for S-sintered monoliths produced at 600 °C at high pressures, before firstly decreasing with increasing temperature and finally, reaching plateau regions of about 3.85 and 4.0 nm for S-sintered monoliths at medium and high pressures and C-sintered monoliths, respectively. Thus, an increase in pore wall thickness, as reported in high-temperature stability studies of SBA-15 powder,^54^ could not be observed for sintered COK-12 monoliths.

Compared to the wall thickness, the wall area, shown in Fig. 6b, allows observing the effects of changing lattice parameter and mesopore size in a larger, 2D environment. Starting off with a wall area of 31.8 nm^2^ for the COK-12 powder, the wall area constantly decreases with increasing temperature for the C-sintered monoliths, enabled by unrestricted shrinkage of the diameter and sufficient time to reach equilibrium during pressureless sintering for 12 h. In comparison, the wall area was preserved to a greater extent when high pressures were applied during S-sintering, restricting both lattice parameter and mesopore shrinkage.

While commonly high specific surface areas are accompanied by enhanced sinterability, for mesoporous materials especially the pore arrangement and a high pore diameter to wall thickness ratio were found to be crucial to strong sintering performance.^60^ For example, finite element modelling yielded higher von Mises stresses and thus, easier mesopore collapse and cracking in cubic systems in comparison to hexagonal systems. The pore diameter to wall thickness ratio of COK-12 is 1.47 or 1.44, as determined using the mesopore size obtained from SAXS or NLDFT, respectively, which is in the range of the ratio for SBA-15 reported with 1.5 ± 0.06.^60^ However, the pore size for the calculation of the ratio for SBA-15 was calculated using the pore size obtained from the Barrett–Joyner–Halenda (BJH) model, which is known to severely underestimate small- and medium-sized mesopores and thus, results in an overestimated pore diameter to thickness ratio.^53,61^ Hence, a lower pore diameter to wall thickness ratio can be postulated for COK-12 in comparison with SBA-15. COK-12's higher resilience towards sintering is furthermore supported by a higher condensation degree of the silica framework within COK-12, which is reported to increase its resistance towards compaction,^26,62^ and the higher temperatures and processing times required to obtain dense structures by S-sintering. To fully eliminate the porosity in SBA-15 structures, temperatures of 900 °C, in between 930 and 980 °C, 1020 °C, and 1040 °C were reported,^22,60,63,64^ being only slightly higher than the 900 °C applied to COK-12 during S- and C-sintering, resulting in porous monoliths. Hence, further parameters affecting the densification, namely pressure, dwell time, and heating rate, were reported to be the same or smaller, ensuring reasonable comparability. For comparison, approaches within this work to densify COK-12 required S-sintering with a slow heating rate of 25 K min^−1^, a long dwell time of 45 min, and temperature and pressure of 1045 °C and 50 MPa, respectively.

The silica peak positions and peak widths of the COK-12 powder and monoliths obtained by SAXS fitting at around 15 nm^−1^ are shown in Fig. S17.† After a slight increase at 600 °C for medium and high pressures, the silica peak positions slightly shift towards lower q-values with increasing temperature, indicating a small increase in the overall silica structure size. Similarly, a decrease in the silica peak width can be observed with increasing temperature, indicating a narrower population of the silica species. To gain a deeper understanding on the atom surroundings in the processed COK-12 with increasing temperature, a series of S-sintered monoliths processed at a pressure of 25 MPa at varying temperatures was measured in total scattering and analyzed by the PDF method. As shown in Fig. S18,† the peak positions at around 1.7, 2.2 and 2.7 Å, which can be associated to the first order pairs of Si–O, O–O, and Si–Si, respectively, remain stationary for all temperatures. However, in comparison to uncompressed amorphous silica, whose Si–O peak position is commonly found at around 1.6 Å, the COK-12 monoliths' peaks appear at higher interatomic distances, whereas the position of the O–O and Si–Si reflections, commonly found in the range of 2.3–3.4 and 2.7–3.1 Å, respectively, are shifted towards lower interatomic distances.^65–68^ Accordingly, the peak shifts might be associated to a pressure-induced increase in the Si–O distances and a decrease in the O–O and Si–Si distances.^66^ Furthermore, as the following peaks are distinguished, a high ordering of SiO_4_ tetrahedra can be assumed.^65^ However, the distinct peak at around 3.4 Å, associated to the second order of the O–O distances, can also be an indication of the evolution of an SiO_5_ network with oxygen edge-sharing atoms in contrast to the SiO_4_ network with oxygen corner-sharing atoms.^66^ While there is no shift in the monoliths' peak positions, the intensity of the Si–O peak increases with increasing processing temperature, signifying a higher probability of occurrence for this atomic pair and a higher coordination number and/or higher ordering.^69,70^ This is in accordance with the small increase of the overall silica structure size and its narrowed population observed by SAXS. Overall, the observed changes in the monoliths' structure size and population as well as bond lengths are minor, which is in agreement with the XRD results shown in Fig. S19,† revealing only marginal differences in the respective short-range order around 20°, whereas the COK-12 monoliths processed by S-and C-sintering remain amorphous up to 50 MPa and 900 °C. Significant structure changes, in the form of enhanced silica structure growth and partial crystallization, can only be observed in the dense sample processed at 50 MPa and a higher temperature of 1045 °C.

**Fig. 7:**
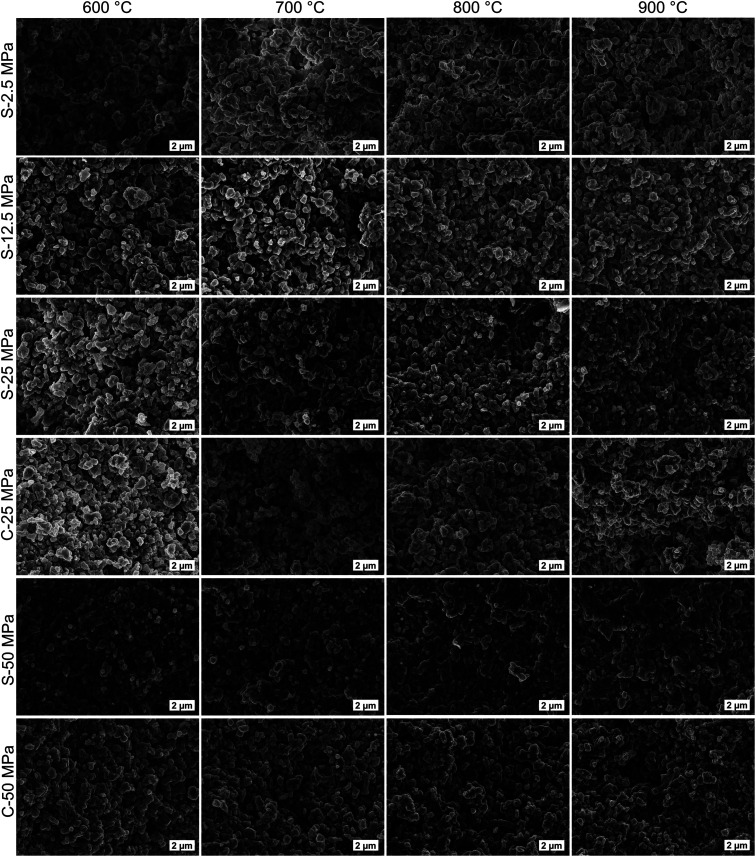
SEM images of the fractured surfaces of S- and C-sintered COK-12 monoliths after mechanical testing.

The macrostructure of the S- and C-sintered monoliths can be appraised from the SEM images in Fig. 7. In addition to the small macropores ascertained by SAXS, macropores not larger than a few micrometers can be observed in the SEM images. The particle shape and size were maintained to a large extent for S-sintered monoliths up to a processing temperature and pressure of 800 °C and 25 MPa, respectively, compare the morphology of the COK-12 powder in Fig. S20.† For higher temperatures and pressures, the presence and dimension of sintering necks, a result of progressing densification, becomes more evident. However, grain growth remains moderate even at high temperatures. This can be attributed to the high heating rate and short dwell time during the SPS process. Surprisingly, sintering necks, densification, and grain coarsening can also hardly be observed for C-sintered monoliths, despite the long sintering time and shrinkage of up to 10.5% in sample diameter. While commonly, the sintering of NPs such as COK-12 is kinetically enhanced due to the high surface area, it can be concluded that the energy provided at 600–900 °C was not enough of a driving force to progress the sintering process adequately. Furthermore, phenomena such as interparticle friction, NP agglomeration, and the pinning action of open pores in the NP structure present additional challenges for the sintering process,^71^ all being applicable to OMS materials such as COK-12. As those phenomena are expected to be counteracted to a certain extent by the application of external pressure during the sintering process, resulting in stronger, porous COK-12 monoliths when produced by S-sintering in comparison to C-sintering as discussed in detail below.

**Fig. 8:**
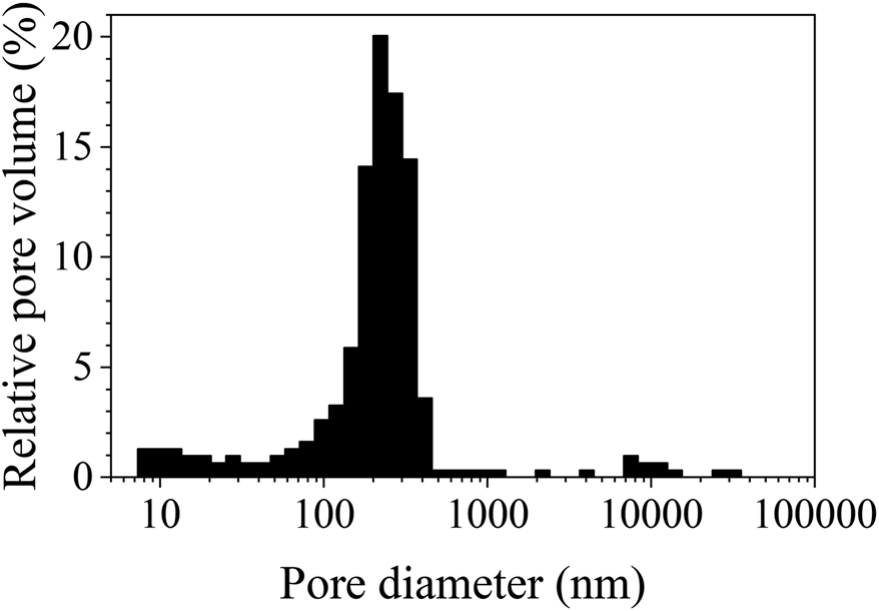
Pore size distribution histogram obtained from mercury intrusion porosimetry data of S-sintered COK-12 monoliths processed at 12.5 MPa and 800 °C.

The pore size distribution histogram obtained from the mercury intrusion porosimetry data on S-sintered monoliths processed at 12.5 MPa and 800 °C is shown in Fig. 8 and reveals information in the small to medium macropore range, in particular, complementing nitrogen sorption, SAXS, and SEM information. The porosimetry data reveals a uniform pore size distribution with a median pore diameter of 322 nm (by volume), suitable for microfiltration applications. Those macropores can be attributed to interparticle cavities as a result of the deliberately incomplete sintering process. Furthermore, a noticeable amount of mesopores were detected at the lower measurement limit of the mercury porosimeter, which is in overall agreement with the nitrogen sorption and SAXS data. Deviations towards the results obtained by other measurement techniques may be ascribed to the somewhat arbitrary nature of the mercury porosimetry results due to the assumption of cylindrical pores and the inability to ascertain the true inner pore size in the presence of wider pore openings.^72^

**Fig. 9:**
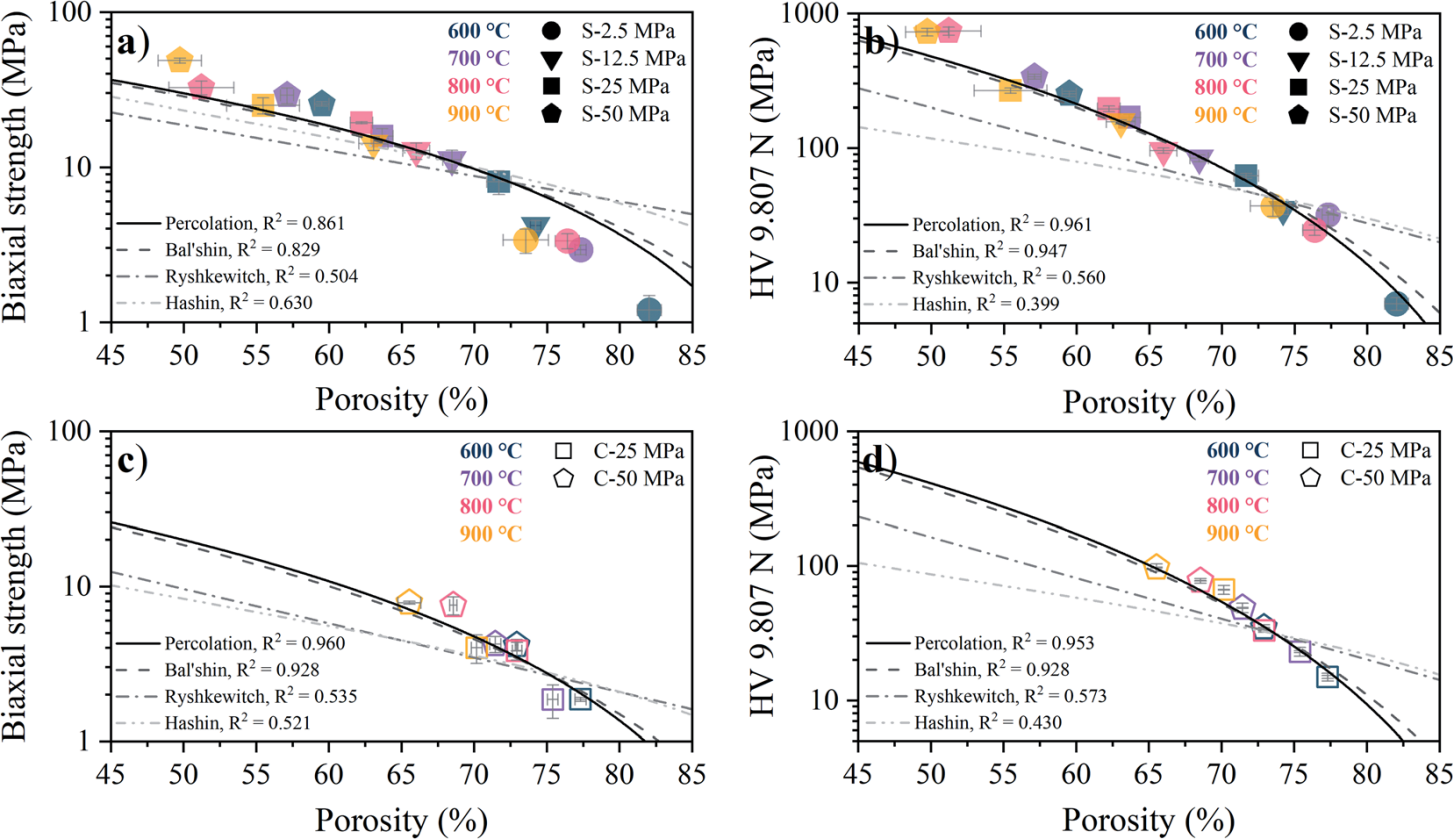
Biaxial strength (a) and (c) and Vickers hardness HV 9.807 N (b) and (d) over porosity for varying sintering temperatures and pressures for S- and C-sintered COK-12 monoliths as well as corresponding Percolation, Bal'shin, Ryshkewitch, and Hashin fits.

The porosity dependence of the biaxial strength obtained from B3B testing and the apparent hardness obtained from Vickers testing for the S- and C-sintered COK-12 monoliths sintered at different temperatures and pressures are shown in Fig. 9. Overall, an expected increase in the mechanical properties with decreasing porosity, concomitant with increasing processing pressure and temperature, can be observed, which can be associated to the elimination of pores and thus, an increase in the effective cross section of the sample. It can be observed that the S-sintered monoliths exhibit 4–8 times higher biaxial strength values and 4–10 times higher Vickers hardness values, reaching up to 49 MPa and 736 MPa HV 9.807 N, respectively, while presenting 4–17% lower porosity in comparison to the C-sintered monoliths at the same processing pressure and temperature.

While no biaxial strength values are yet reported in the literature for S-sintered samples, the Vickers hardness of SBA-15 was reported with 92–250 MPa for S-sintering temperatures of 600–900 °C at a processing pressure of 10.6 MPa and resulting porosities of 75–65% at an indentation load of 100 g.^22^ These values are slightly higher than those presented for COK-12 at comparable processing conditions, compare Fig. 9b. On the contrary, SBA-15 based silicon-boron-carbon-nitrogen monoliths yielded Vickers hardness values of 16.7–21.8 MPa at an indentation load of 30 N for SPS temperatures of 800–900 °C, a pressure of 16 MPa, and resulting porosities of only 69–65%, being significantly lower in comparison to the COK-12. Those differences can be attributed to the indentation size effect, which describes decreasing hardness with increasing test load, wherefore the application of standards is encouraged.^73^

For a fixed processing pressure, the porosity was found to decrease in a linear manner with increasing temperature, as depicted in Fig. 1b. The porosities of the C-sintered monoliths processed at 25 and 50 MPa can be located between the porosities of the S-sintered monoliths processed at 2.5 and 12.5 MPa. While also the dependence of the porosity on the mechanical properties is often considered to be linear, usually due to the lack of data over a sufficient porosity range, it is known to be non-linear.^37^ Hence, Fig. 9 includes the fitting of the mechanical strength data over porosity with the non-linear relationships according to the Bal'shin, Ryshkewitch, and Hashin model listed in eqn (1)–(3), using *σ*_0_ = 124.3 MPa and HV_0_ = 5360 MPa. Furthermore, the percolation law from eqn (4) was applied, which interestingly has not been proposed yet to fit the mechanical properties over porosity, whereas however, the power law expression by Rzhevsky and Novik, a particular case of the percolation law, is commonly applied.^37^ The fitting of the Rzhevsky and Novik as well as the Eudier model were not meaningful as they yielded a local hardness minimum at a porosity of around 78% or include an intrinsic porosity limit of 75%, respectively. While fitting of the Hashin and Ryshkewitch models did not yield satisfactory regression coefficients for the processed COK-12, although the Ryshkewitch model, in particular, is commonly applied to assess and extrapolate the mechanical properties for S-sintered samples with reasonable results for metallic titanium foams^74^ and only mediocre estimates for ceramic silica and silica composites,^22,75^ the percolation law and the Bal'shin model yielded better fittings for the COK-12 monoliths with regression coefficients of 0.865–0.964 and 0.829–0.947, respectively. The similar regression coefficients can be attributed to the fact that the Bal'shin model is a particular case of the percolation law. The higher fitting values for the C-sintering and the slightly better fit of the percolation law can be attributed to the lower porosity range of the data and the additional parameter *ε*_M_, respectively. However, even without this information, reasonable fitting is possible using the Bal'shin model. The resulting percolation fitting parameters are 1.9 and 2.5 for the biaxial strength and 3.2 and 3.4 for the Vickers hardness for S- and C-sintered monoliths, respectively. A summary of all regression coefficients and corresponding fitting parameters can be found in Tables S2 and S3.† The fitting parameter for the percolation law, also referred to as the characteristic exponent, describes the change of the property towards *ε*_M_.^76^ Thus, the higher fitting parameters of the C-sintered monoliths can be associated to a faster loss in the mechanical properties with increasing porosity in comparison to the S-sintered monoliths. Thus, it can be assumed that the sintering necks between the COK-12 particles are more resilient when processed with S- in comparison to C-sintering, even considering that different processing pressures and temperatures are needed to achieve comparable porosities. The difference in the fitting parameter between the S- and C-sintering is more distinct for the biaxial strength than for the hardness. This can be associated to the additional tensile portion of the B3B test in comparison to the hardness testing, which is performed in compression mode.

**Fig. 10:**
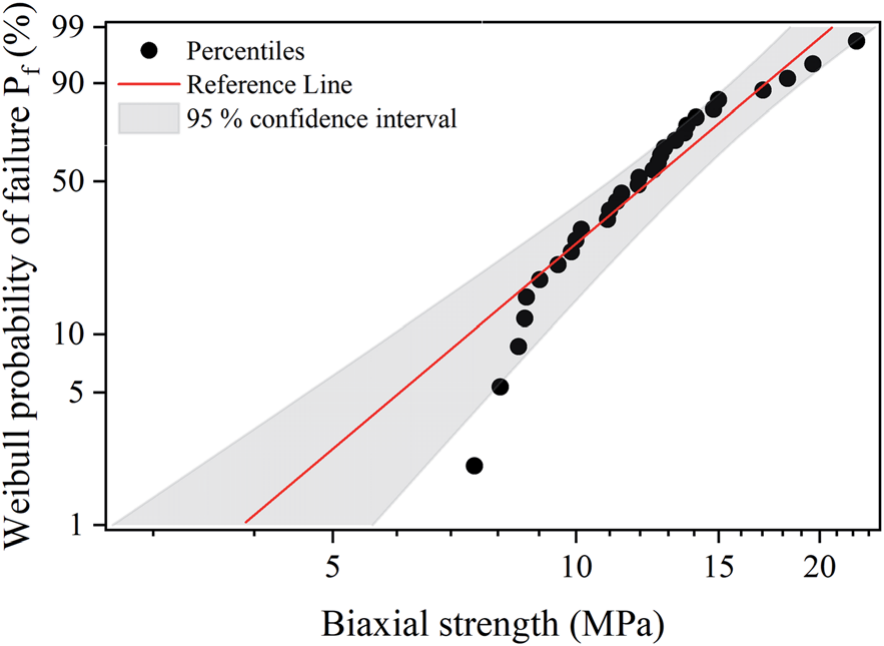
Weibull probability plot for biaxial strength values obtained by B3B test on S-sintered monoliths processed at 800 °C and 12.5 MPa.

To predict the probability of failure of S-sintered COK-12 monoliths, a Weibull analysis was performed on biaxial strength data for S-sintered monoliths processed at 12.5 MPa and at 800 °C with an apparent porosity of 66%, whose results are shown in Fig. 10. The results reveal maximum likelihood estimates of the Weibull characteristic strength 
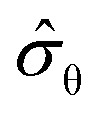
 and Weibull modulus *m̂* of 13.7 MPa and 3.7, respectively. With an average standard deviation of 1.2 MPa within the three monoliths obtained per S-sintering run, it can be concluded that the parallel configuration of the graphite die works reliably. The obtained Weibull modulus is lower than the common range of 5–10, which is usually expected for engineered ceramics.^77^ A low Weibull modulus is reported to be associated with high porosities and pore volumes in general, but in particular with high interparticle porosities and wide pore size distributions, which both is applicable to the sintered COK-12, whose hierarchical pore structure comes along with an increased probability for crack growth.^78–80^ It should be highlighted that the latter applies only for tests performed in bending mode, as crack propagation is different in compressive mode. The data points displayed in Fig. 10 show a P-type behavior, *i.e.*, a positive deviation from the well-known linear behavior, which occurs when the lower tail of the failure stresses deviates from the fitted line towards the mean failure stress. P-type behavior, also referred to as three parameter distribution, is reported to be associated with pore pairs favoring enhanced stress localization in brittle porous materials and to be related to internal compressive stresses in general, such as surface stresses.^81,82^

**Fig. 11:**
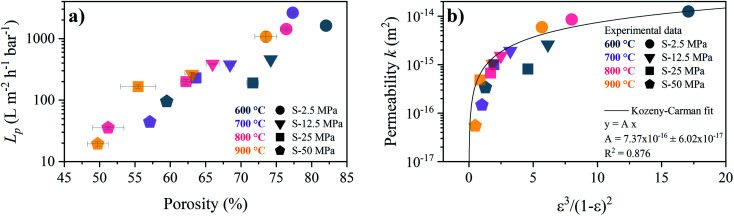
Relation between porosity and pure water flow behavior for S-sintered monoliths. (a) water permeance *L*_p_ (b) permeability *k* and corresponding Kozeny–Carman fit.

The porosity dependence of the pure water permeance for the S-sintered monoliths is shown in Fig. 11a. The pure water fluxes at different pressures and the corresponding linear fits, from which the permeance was derived, can be found in Fig. S21.† As expected, higher porosities resulted in higher water permeance. The monoliths produced at the lowest pressure of 2.5 MPa and highest pressure of 50 MPa show permeances above 1000 L m^2^ h^−1^ bar^−1^ and below 100 L m^2^ h^−1^ bar^−1^, respectively. For the lowest and highest pressure, a distinct permeance deterioration can be observed with increasing temperature. In comparison, for moderate pressures of 12.5 MPa and 25 MPa the permeance scattering with temperature is markedly less pronounced while spreading over a wider porosity range. As the macropore volume can be expected to have a major influence on the mass transport, relative to the total porosity including also the micro- and mesopore volume, the dependence of the permeance on the estimated macropore volume is shown in Fig. S22.† In comparison to the dependence of the porosity, a sharper increase in the permeance can be observed with increasing macropore volume, in particular for low macropore volumes up to 0.36 cm^3^ g^−1^, predominantly corresponding to monoliths processed at the highest pressure of 50 MPa, before reaching a maximum at 1.82 cm^3^ g^−1^ for the lowest processing pressure and temperature of 2.5 MPa and 600 °C. At the same time, the percentage of the macropore volume from the total pore volume increases from 51% to 62% and finally to 83%, respectively, enabling reasonable water permeance. While all S-sintered monoliths withstood applied pressures of up to 7 bar in the experiments without being damaged, it was not possible to determine the water flow behavior of the C-sintered COK-12 monoliths due to collapse prior to or at pressurization of 1 bar.

As the monolith thickness varied significantly with the processing pressure and temperature, the permeability *k*, calculated from eqn (9), was obtained as an intrinsic, thickness-independent parameter and plotted against the porosity term of the Kozeny–Carman equation in Fig. 11b. During the fitting of the Kozeny–Carman equation, an outlier exhibiting a studentized residual larger than three, namely the S-sintered monolith processed at 2.5 MPa and 700 °C, was removed from the data for fitting purposes. Fitting with the complete data and the list of corresponding studentized residuals can be found in Fig. S23 and Table S4,† respectively. The permeability values were found to be in the range of 5.5 × 10^−17^ to 1.3 × 10^−14^ m^2^. Fitting the Kozeny–Carman equation resulted in a reasonable regression coefficient of 0.876 and yielded a Kozeny–Carman parameter *A* of 7.37 × 10^−16^. Deviations from the model can be explained by simplifications and assumptions that are violated, such as isotropic porous media and structure consisting of well-packed spheres of a specific size opposing uniaxial pressing and consolidation of plate-like particles. However, as other models are based on considerably more assumptions, the Kozeny–Carman equation seems to be the best expression available for modelling flow through porous media.^83^ Using the obtained Kozeny–Carman parameter, compare eqn (10), the common range of *c*_0_ being 1/6–1/2, and a specific surface area *S*_0_ with respect to the unit volume of the solid matrix of 6.45 × 10^7^ m^2^ m^−3^ by utilizing a COK-12 powder density of 0.1 g cm^−3^, the tortuosity factor *τ* can be estimated.^62,84^ Considering the definition of *τ* = (*L*/*L*_e_)^2^ < 1 with *L* and *L*_e_ being the length of the porous medium and effective flow path length, respectively, *τ* was determined to 0.81–1. Transposing the tortuosity factor into the more common parameter tortuosity *L*_e_/*L* yields values in the range of 1–1.11, which can be considered underestimated, because on the one hand the monoliths do not exhibit straight-through pores, compare Fig. 7, and on the other hand tortuosity values for porous membranes and unconsolidated mesoporous silica materials are typically found in the range of 1.5–5 and 1.4–4.2, respectively.^85–87^ Furthermore, it is worth noticing that the tortuosity values were obtained from fitting of monoliths among a wide porosity range, so that changes in the tortuosity with the processing parameters pressure and temperature were not considered, which, however, can reasonably be assumed.

**Fig. 12:**
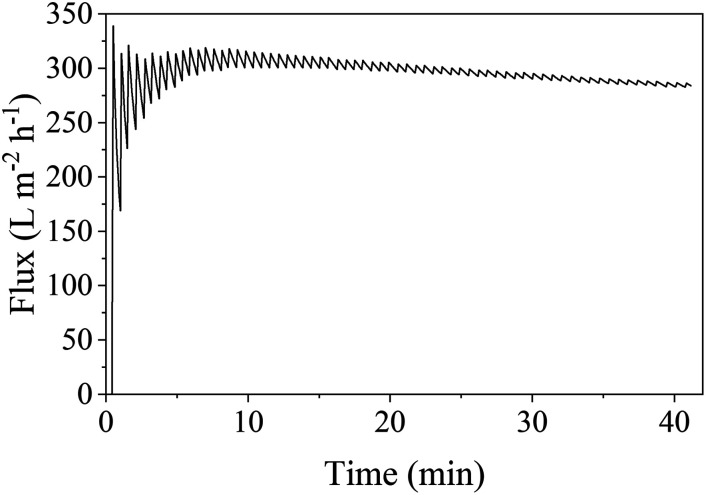
Time-dependent flux during the filtration of surfactant-stabilized oil in water emulsion with 100 mg L^−1^ oil at a transmembrane pressure of 1 bar through an S-sintered monolith processed at 12.5 MPa and 800 °C. The initial COD of 366 mg L^−1^ could be reduced by 90% to 35 mg L^−1^.

The oil droplet size distribution of the surfactant-stabilized oil in water emulsion used for the filtration experiment is shown in Fig. S24† and reveals a mean oil droplet size of 25 μm and *d*_90_ and *d*_10_ values of 47.9 and 2.3 μm, respectively. Filtration of this emulsion using an S-sintered monolith processed at 12.5 MPa and 800 °C, exhibiting a beneficial pure water permeance to porosity ratio, at a transmembrane pressure of 1 bar resulted in a flux of about 298 L m^−2^ h^−1^, as depicted in Fig. 12. While the flux declined by 20% in comparison to the pure water flux of 373 L m^−2^ h^−1^, it remained stable along the filtration time of 40 min, which allows concluding a reasonable resistance towards fouling, which can be attributed to the hydrophilic character of the COK-12 due to the presence of surface hydroxyl groups, which inhibit the adherence of foulants to the surface by steric repulsion as commonly introduced by surface modification of polymeric membranes.^88–91^ During filtration, the COD was reduced by 90%, meaning from 366 to 35 mg L^−1^. Thus, the smallest diameter retained can be estimated to correspond to the *d*_10_ value of 2.3 μm. The overall performance, including the required pressure, flux, and retained diameter, classifies the COK-12 monoliths within the microfiltration membrane category. The higher condensation degree and thicker silica walls in comparison to other mesoporous silica materials such as SBA-15 facilitate a higher stability for COK-12 in an aqueous environment.^26^ While high pH cleaning is rather unsuitable for the regeneration of oil-loaded silica surfaces due to limited chemical resistance, the monolith's resistance towards ultrasonic and thermal cleaning can potentially be utilized to enhance the filtration process as well as an environmentally friendly cleaning method.^92^ The oil in water filtration of the S-sintered COK-12 monolith is more effective in comparison to S-sintered monoliths produced from volcanic shirasu balloon, which exhibit lower porosities and larger pores at comparable processing parameters.^93^ Further S-sintered materials such as diatomite were suggested to be suitable for water purification applications.^94^ Furthermore, the produced COK-12 monoliths may present additional benefits, such as molecule capture, due to their hierarchical pore structure from the macro-to the meso-/micropore scale. Their properties also make them interesting for applications in HPLC separation and membrane reactors, as catalysis support, host system for controlled drug delivery, or for tissue engineering.^95^

## 4 Conclusions

Lightweight, hierarchically porous monoliths were processed from OMS COK-12 by SPS (S-sintering) using a custom graphite multi-sample die in a parallel configuration, thoroughly examined with respect to their structural, mechanical, and water permeability properties, and compared to conventionally (C-) sintered monoliths.

Data fitting with a customized SAXS model allowed to examine the influence of the processing parameters on the lattice parameter, micro-/, meso-, and macropore sizes, as well as silica population parameters over a wide q-range, making it interesting as a multi-scale method bridging nitrogen sorption and mercury intrusion measurements. Overall, it was found that for both sintering methods the temperature has a more pronounced effect on the structural parameters than the pressure. Due to the simultaneous application of pressure and temperature, the reduction in porosity, specific surface area, and pore volume was more pronounced in S-sintered monoliths prepared at comparable processing conditions, however, becoming negligible for high processing temperatures. In contrast, the distinct decrease in the mesopore size, micropore volume, and lattice parameter in C-sintered monoliths was attenuated for the S-sintered monoliths due to the restricted particle movement and shorter processing times, resulting in a higher preservation of wall thickness and wall area for the latter. At the same time, higher stress-induced deviations from the theoretical lattice were revealed for the S-sintered monoliths. Overall, COK-12's superior resistance towards sintering in comparison to SBA-15 could be ascribed to COK-12's lower pore diameter to wall thickness ratio and higher silica condensation degree.

The mechanical properties were found to follow the percolation law. Thereby, the S-sintered monoliths showed higher mechanical properties than the C-sintered monoliths at comparable porosities. While the difference was small for the Vickers hardness, it was higher for the B3B biaxial strength data, which can be attributed to the more demanding, biaxial character of the test, emphasizing the higher bending stability for S-sintered monoliths, making them more interesting for load-bearing applications.

In contrast to C-sintered monoliths, S-sintered monoliths could withstand ultrasonic cleaning and pressures of 7 bar in a dead-end water filtration setup, yielding high permeances and permeabilities while following the Kozeny–Carman relationship. A selected S-sintered monolith, utilized for the filtration of a surfactant-stabilized oil in water emulsion, presented a high separation efficiency and a stable flux, indicating a reasonable resistance towards fouling, likely due to COK-12's surface hydroxyl groups.

Future work might include the generation of a mesopore gradient throughout the monoliths thickness by layer-wise deployment of COK-12 powder with different pore sizes or shapes. Thereby, also larger macropores could be introduced through a secondary sacrificial templating agent, such as sodium chloride crystals. Furthermore, P123's potential on the mesopore and lattice stabilization, when only removed after the S-sintering process, might be studied. In addition, the large accessible surface area and pore volume might be used for the functionalization with various functional groups. Overall, their structural and mechanical properties along with their development potentialities make the hierarchically porous and mechanically stable COK-12 monoliths promising for applications in separation and catalysis.

## Author contributions

L. M. H. conceptualization, methodology, formal analysis, investigation, data curation, writing – original draft, visualization, project administration; J. T. M. investigation, writing – review & editing; G. J. S. conceptualization, methodology, software, validation, formal analysis, data curation, writing – original draft, visualization; B. R. P. methodology, software, writing – review & editing; M. F. B. conceptualization, methodology, writing – review & editing; supervision; J. S. formal analysis, writing – review & editing; A. G. conceptualization, resources, writing – review & editing, supervision, funding acquisition; U. S. conceptualization, writing – review & editing, supervision.

## Conflicts of interest

There are no conflicts to declare.

## Supplementary Material

NA-004-D2NA00368F-s001
