# Supplementary material for: Hierarchically porous and mechanically stable monoliths from ordered mesoporous silica and their water filtration potential
Source: Nanoscale Adv. 2022 Aug 15;4(18):3892–908. doi: 10.1039/d2na00368f (PMC9470055; doi:10.1039/d2na00368f)
Supplement: NA-004-D2NA00368F-s001 [file NA-004-D2NA00368F-s001.pdf]

Electronic Supplementary Material (ESI) for Nanoscale Advances.
This journal is © The Royal Society of Chemistry 2022

Supporting information

Hierarchically porous and mechanically stable monoliths from ordered mesoporous silica and their water filtration potential

Laura M. Henning[a*], Julian T. Müller[a], Glen J. Smales[b*], Brian R. Pauw[b], Johannes Schmidt[c], Maged F. Bekheet[a], Aleksander Gurlo[a#], Ulla Simon[a#]

[a] Technische Universität Berlin, Faculty III Process Sciences, Institute of Material Science and Technology, Chair of Advanced Ceramic Materials, Straße des 17. Juni 135, 10623 Berlin, Germany

[b] Bundesanstalt für Materialforschung und -prüfung (BAM), Division 6.5 – Polymers in Life Sciences and Nanotechnology, Unter den Eichen 87, 12205 Berlin, Germany

[c] Technische Universität Berlin, Faculty II Mathematics and Natural Sciences, Institute of Chemistry, Chair of Functional Materials, Straße des 17. Juni 135, 10623 Berlin, Germany

* corresponding authors. Contact details: L.M.H. e-mail address laura.m.henning@ceramics.tu-berlin.de, telephone: +49 (0) 30 314 70483; G.J.S. e-mail address glen-jacob.smales@bam.de, telephone: +49 (0) 30 8104 3314

[#] equal contribution

Experimental

Spark plasma sintering

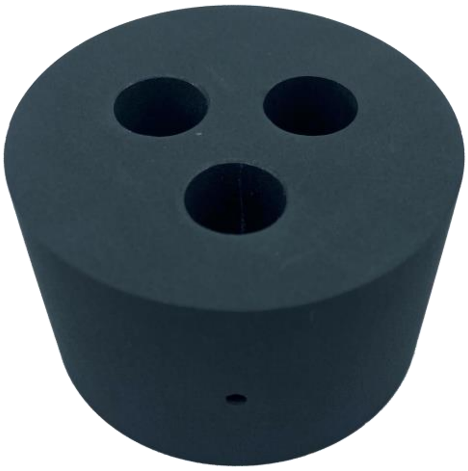

Figure S1. Photograph of one of the custom, cylindrical three-sample graphite dies used for spark plasma sintering (SPS, S-sintering) with a height of 30 mm, outer diameter of 60 mm, and inner diameter of 3x ⌀10 mm.

SAXS/ WAXS

On modelling and simulations

X-ray scattering is used to quantify nanoscale structures, with small-angle X-ray scattering (SAXS) used for structures typically in the 1–350 nm range, and wide-angle X-ray scattering (WAXS) used for probing interatomic and intermolecular distances. Through their combination, i.e., by collecting X-ray scattering data with a large q-range, a wealth of structural

information can be obtained. For complex multi-scale structures, such as those present in COK-12, the data is rich in features arising from the different structural elements, including the hexagonally space mesopores, micropores and macropores, as well as scattering from the surface of large powder grains. The interpretation of such data, therefore, is reliant on the composition of a well-founded multicomponent analysis model where the individual scattering features arising from the many–occasionally overlapping or interacting–structures can be described. To describe the scattering for COK-12, one such multicomponent analysis model was constructed in the SASfit small-angle scattering data analysis package.[1]

Given the complexity of the model, it is prudent that the origin of the scattering features in the data is well understood. To this end, we validated the analysis model choices by a systematic study of simulated datasets of a model COK-12 structure, similar to our approach in.[2,3] For COK-12, we created a real-space structural model using the OpenSCAD 3D modelling software[4] consisting of a 100 nm high hexagonal array of cylinders with optional (Gaussian) dispersity in cylinder radius as well as their lattice positions. Using this, we generated a series of 3D structures in the STereo Lithography (STL) file format, which the SPONGE then used to simulate the corresponding X-ray scattering pattern.[5] By systematically varying structural parameters in the real-space model and observing their effect on the scattering patterns, we could verify the links between these two. This, in turn, allowed us to validate the origin of particular data features and substantiate their use in the data analysis model. Likewise, the SASfit model can also be decomposed to reveal the underlying components and the impact they have on the data.

Simulation findings

First, the effect of pore size on a hexagonal lattice was investigated, in particular to explain the variations in hexagonal peak heights. This investigation employs a combination of a cylindrical form factor and a hexagonal structure factor in SASfit (**Figure S2a** and **S3**), and appropriate 3D models (**Figure S2c**) for the SPONGE (**Figure S2b**). These simulations show that the hexagonal structure factor peaks strongly interact with the cylinder form factor and is most prominently visible by comparing the (11) and (20) peak intensities. Hence, it can be concluded that the (11):(20) peak intensity ratio is a result of the interplay (convolution) between the hexagonal structure factor and the minima of the cylindrical form factor. As such, it is dependent on both the lattice spacing as well as the pore radius.

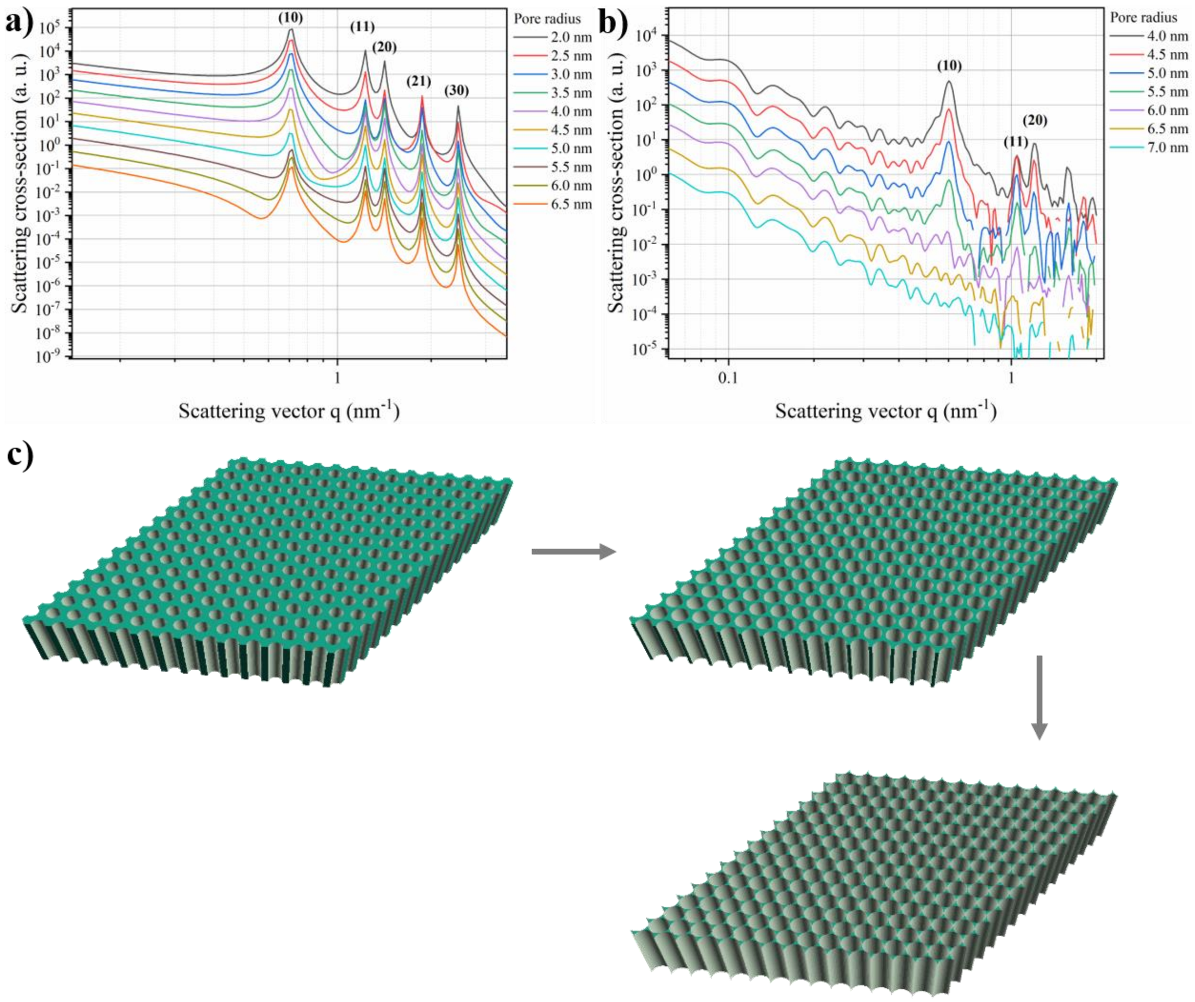

Figure S2. Simulations of SAXS curves using a cylindrical form factor and a hexagonal structure factor with a lattice constant of 10.24 nm (a). The cylindrical contribution has parameters of the length set to 500 nm and radial polydispersity set to ca. 10 %. (b) Scattering curves simulated from 3D models (c) using the SPONGE, with varying pore radii and on a hexagonal lattice. For the 3D models shown (c), the lattice parameter is set at 12 nm, with pore radii of 4, 5 and 6 nm shown.

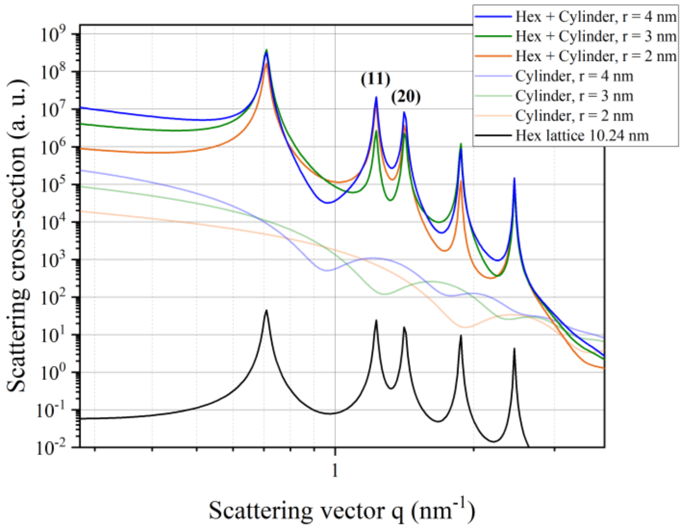

Figure S3. Simulation of a hexagonal lattice structure factor (black) and cylindrical form factors with radii of 2, 3 and 4 nm, alongside the resulting scattering curves from combining (multiplying) the hexagonal structure factor with the form factor appropriate for the pore dimensions.

With the SPONGE simulations (**Figure S2b)** a special case is observed when the pore radius approaches a value of half the lattice parameter (6 nm for these simulations, with a hexagonal pore spacing of 12 nm). In this case, the (10) reflections are drastically reduced in intensity, with the entire set of hexagonal structure peaks disappearing with larger pore sizes as would be expected when a pore structure engulfs the lattice. This phenomenon is, however, not observed in the classical model which does not account for such physical interactions between neighbors (**Figure S2a**). With the classical approach to modelling, it is possible to simulate "scattering" from physically unrealistic pore structures, such as structures with pore diameters in excess of the hexagonal lattice dimensions. Hence, care should be taken when using classical models to ensure that the physical structure it purports to describe is still within the realistic realm.

Secondly, the polydispersity of the cylinder radii was varied using both SASfit and SPONGE methods (**Figure S4a-b)**. From simulations using the SPONGE, the contributions to the scattering from hexagonal lattice starts to reduce significantly at higher levels of polydispersity, though this is not observed in the classical model (**Figure S4a**). Thus, a similar discord between a realistic structure and a classically modelled structure can be demonstrated, reinforcing the previous conclusion that realistic constraints should be separately considered for classical models, and that a cross-check with a SPONGE-like simulation is not an excessive luxury.

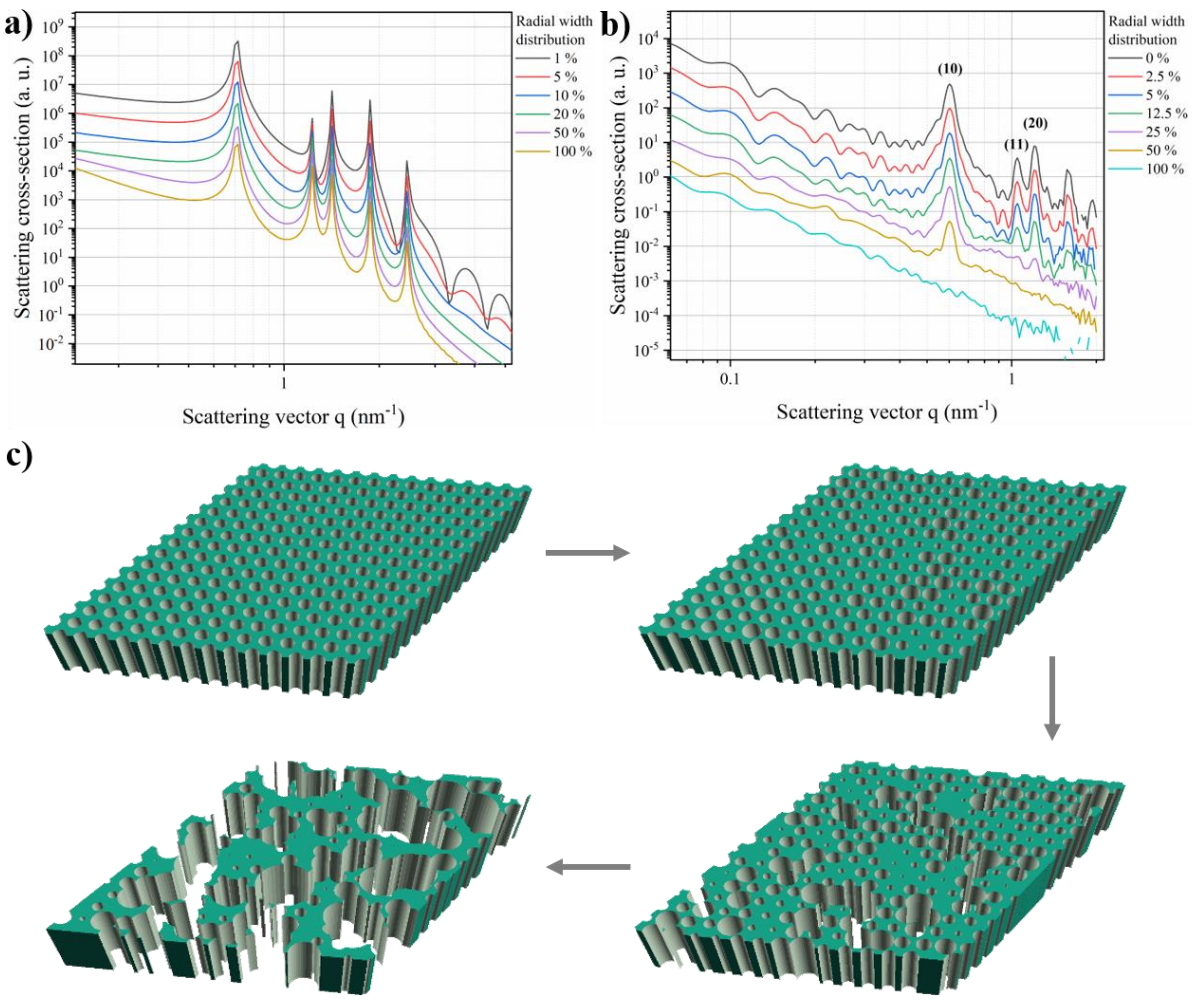

Figure S4. (a) Simulations of SAXS curves using a cylindrical form factor and a hexagonal structure factor with a lattice constant of 10.24 nm. The cylindrical contribution has the length fixed to 500 nm and a radius of 3.1 nm with varying polydispersity in the radial dimension. (b) Scattering curves simulated from 100 nm high 3D models using the SPONGE, with the pore radius set to 4 nm and varying radial polydispersity on a 12 nm-spaced hexagonal lattice. (c) The 3D models, here shown with the Gaussian radial distribution width σ^2 set to 0, 12.5, 50 and 100 % respectively.

A third series explored the effect of positional deviations from the hexagonal lattice positions (**Figure S5**). Here, both simulations show similar trends: as the positional inaccuracy increases, the hexagonal reflections become less well defined, and the normal cylindrical form factor remains. This is in accordance with the known behavior of ordered structures.

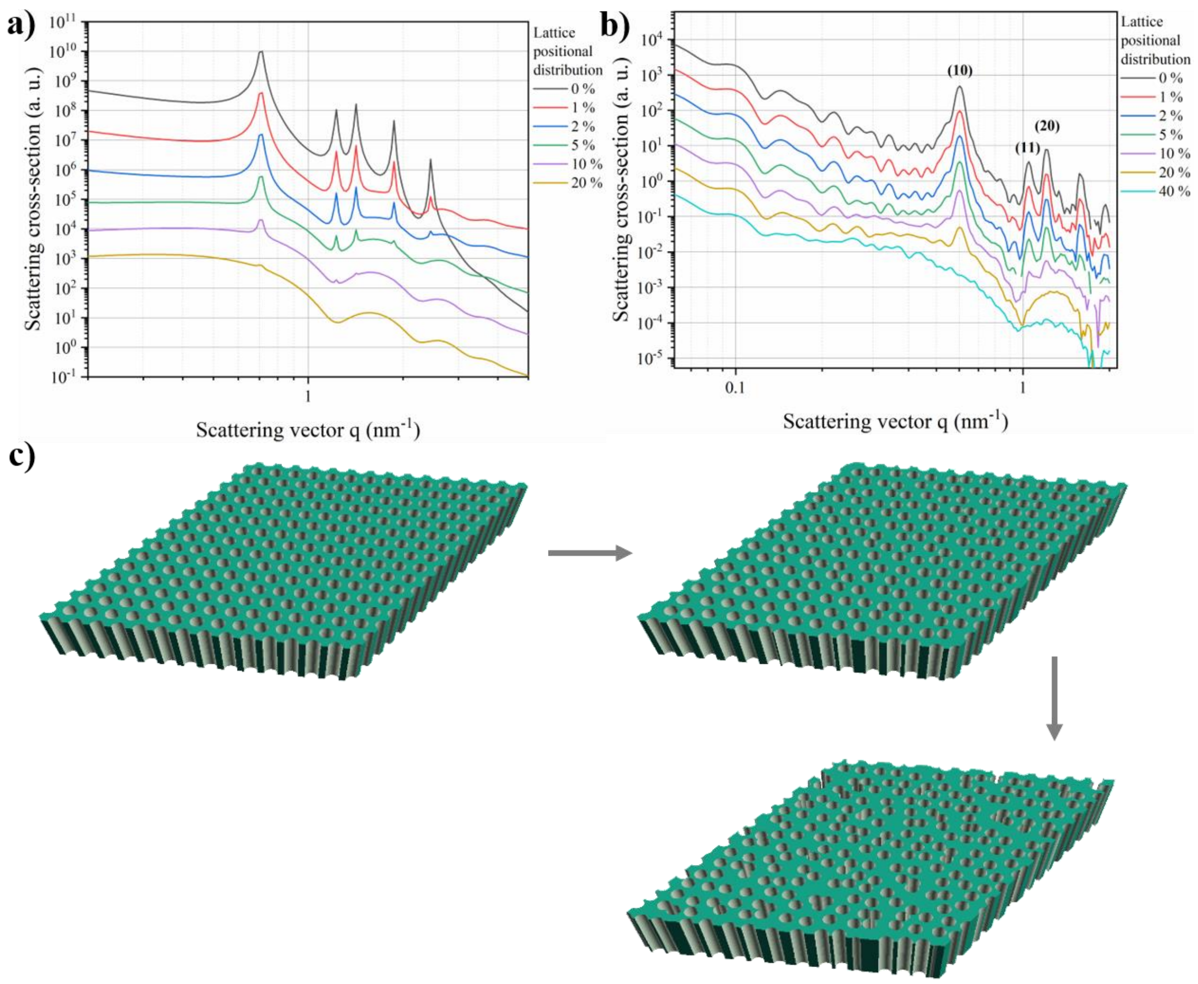

Figure S5. (a) Simulations of SAXS curves using a cylindrical form factor and a hexagonal structure factor with a lattice constant of 10.24 nm. The cylindrical contribution has parameters of the length set to 500 nm and a radius of 3.1 nm with the lattice positional distribution varying. (b) Scattering curves simulated from 3D models (c) using the SPONGE, with varying lattice positional distributions on a 12 nm hexagonal lattice, with the pore radius set to 4 nm. For the 3D models shown (c), the lattice positional distributions are set to 0, 15 and 50 %.

One final consideration to make is how the scattering from micropores can contribute to the scattering from the mesopores (**Figure S6**). The presence of micropores/channels connecting mesopores through the wall of the hexagonal lattice can be modeled equally well using a spherical form factor or a cylindrical form factor. For both, the micropore radius is obtained from a fit of the high-q data, in case of the cylinder, the cylinder length is fixed to the wall thickness obtained from the lattice parameter and mesopore size. Modelling the micropores either as spheres or cylinders has little effect on the observed (11):(20) peak intensity ratio.

These results highlight the importance of collecting a wide q-range for such hierarchical systems, so that information on the interplay of different pore structures can be elucidated and exploited.

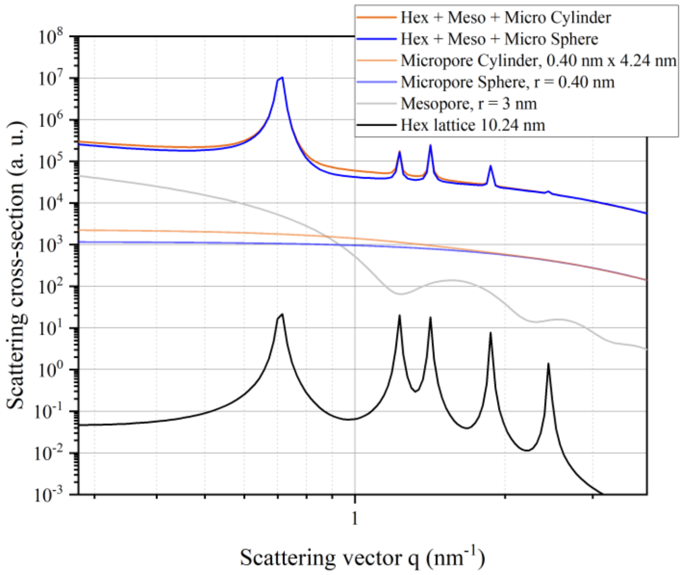

Figure S6. Simulation of scattering curves (orange and blue), consisting of a combination of the following components: hexagonal lattice structure factor (black) multiplied with cylindrical form factor with radii of 3 nm (grey) to represent mesopores. Added to this are either simulations of both cylindrical (light orange) or spherical (light blue) form factors with radii of 0.4 nm to represent scattering from the micropores.

SASfit analysis model

A multicomponent model was constructed using SASfit which is a) able to describe all measured datasets with a consistent model, and b) is able to describe the variations detailed above. The model adds five contributions (adding implies that the components do not constructively interfere):

$$I(q) = I_{BKG}(q) + I_{HEX}(q) + I_{Pore\ large}(q) + I_{Pore\ small}(q) + I_{Silica}(q)$$

Where q (nm^{-1}) is the scattering vector, defined as $q = \frac{4\pi \cdot sin\theta}{\lambda}$, where λ (nm) is the radiation wavelength and with a scattering angle of 2θ.

In the model shown above (also see **Figure S7**), $I_{BKG}(q)$ consists of a flat background contribution and a Porod slope $I \propto q^{-4}$ to account for scattering coming from the surface of large grains. $I_{Hex}(q)$ is the contribution from the hexagonal array of cylindrical channels and is described in detail below. $I_{Pore\ large}(q)$ describes a minor amount of low-q scattering resulting from large macropores found within the COK-12 structures, and is implemented using

a spherical form factor with a log-normal size distribution. $I_{Pore\ small}(q)$ describes a large amount of small pore structures observed at high q, and its origins and implementation are further detailed below. Finally, $I_{Silica}(q)$ is used to describe the broad hump observed in the wide-angle scattering that arises from the intermolecular distance of silica, using a Lorentzian peak function. When using this model, all scattering length densities (SLDs) were set to $\Delta SLD = SLD_{Silica} - SLD_{pore}$.

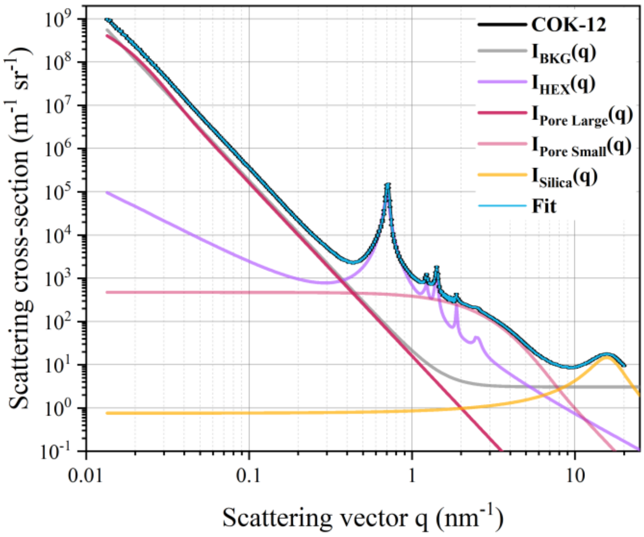

Figure S7. Exemplary scattering curve from COK-12 powder and the subsequent fit, with its individual contributions.

In detail, $I_{Hex}(q)$ is composed of a cylindrical form factor coupled with the HEX-iso-Lorentzian structure factor. The scattering curves from each sample was first analysed with respect to the peak intensity ratio of the (11) and (20) reflections, to obtain an estimate for the cylindrical pore radius, asuming a practical polydispersity of ca. 10 % (the polydispersity was fixed as too little information exists in the data to reliably extract this parameter). The cylindrical form factor also assumes that the length of the cylindrical pores is greater than that of the maximum observable limit of the SAXS measurements (ca. 350 nm) and are therefore fixed to 500 nm.

$I_{Pore\ small}(q)$, describes the micropore structures, found within the samples, also referred to as P2 micropores. These are said to originate from the hydrophilic polyethylene oxide tails of the triblock copolymer, creating intrawall porosity upon their removal. The contribution from these pores is easily observed for the unprocessed COK-12 sample, and decreases significantly when samples undergo S- or C-sintering. The working hypothesis here is that the intrawall pores largely collapse upon the application of high temperature and pressure to the samples. The above five-contribution model describes samples processed at 800 and 900 °C well, but not

those at 600 and 700 °C. For these samples, an additional micropore structure, $I_{Pore\ small\ 2}(q)$ must be added to the model:

$$I(q) = I_{BKG}(q) + I_{HEX}(q) + I_{Pore\ large}(q) + I_{Pore\ small}(q) + I_{Pore\ small\ 2}(q) + I_{Silica}(q)$$

This additional pore structure (**Figure S8**) is of a similar size range as that of the hydrophilic polyethylene oxide tails of the triblock copolymer. One possible explanation for the need to add a second micropore structure, also referred to as P1 micropores, is that there are (at least) two pore collapse processes that occur in these samples, from axially and radially-loaded pores in the packed powders. Then, grains containing pores perpendicular to the axis of applied pressure and grains with pores aligned with the axis of applied pressure could be affected in different ways. Another equally valid explanation could be the formation of intraparticle cracks, which emerge upon processing, yet vanish again at higher temperatures due to enhanced micropore collapse at higher temperatures. Another likely possibility is that the size distribution of the micropores is skewed and, hence, not well described by a single standard distribution function. All we can conclude is that the pore size distribution of these small pores is affected greatly by the processing of the COK-12.

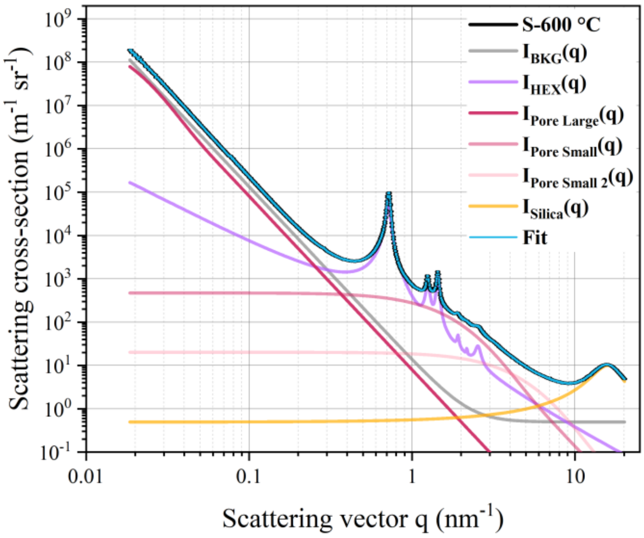

Figure S8. Exemplary scattering curve from a COK-12 monoliths processed at 600 °C and the subsequent fit to a model with two small pore contributions, with its individual contributions decomposed.

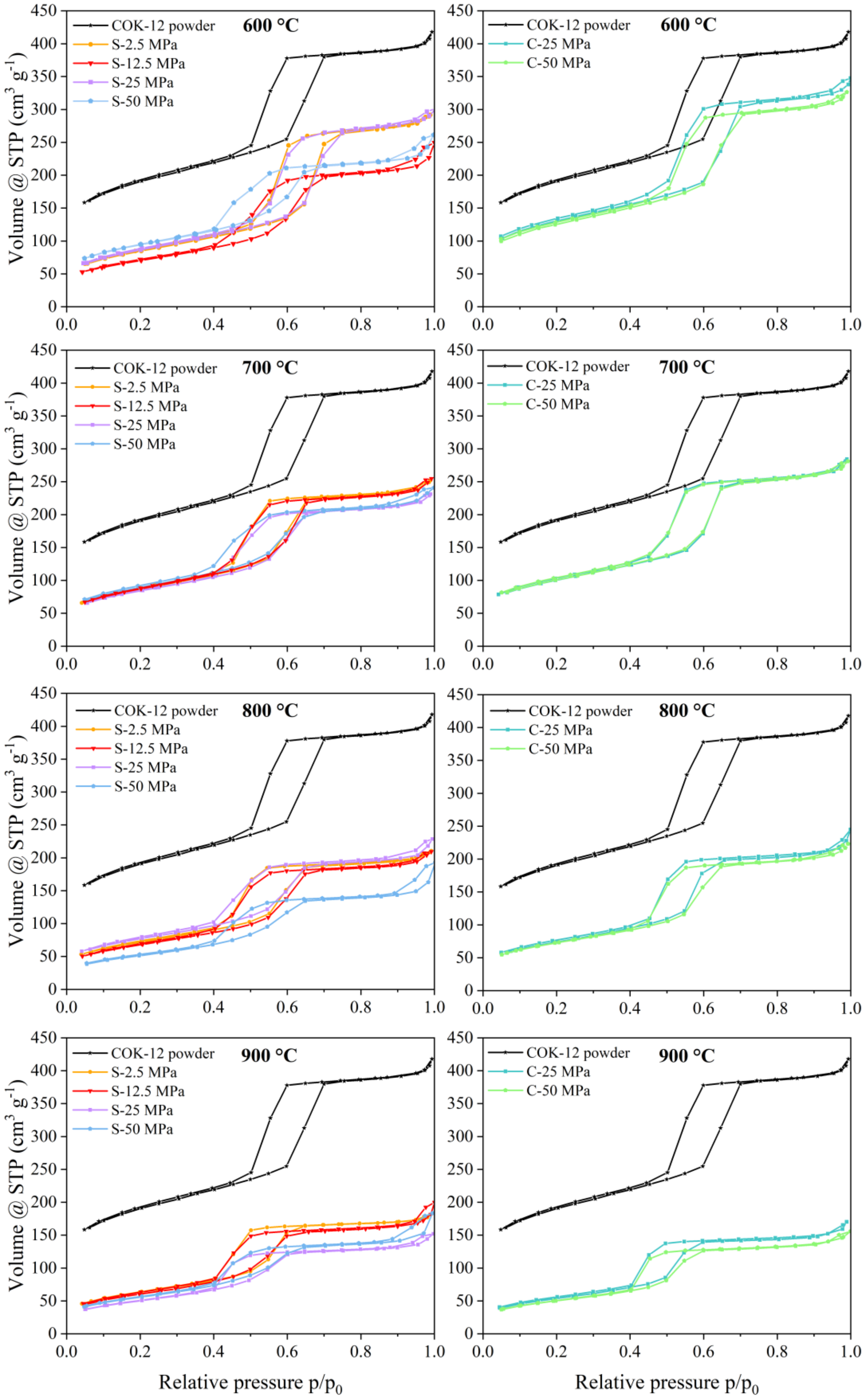

Figure S9. Nitrogen sorption isotherms of COK-12 powder and spark plasma sintered (SPS, S-sintered, left) and conventionally (C-sintered, right) COK-12 monoliths processed at pressures of 2.5–50 MPa and temperatures of 600–900 °C.

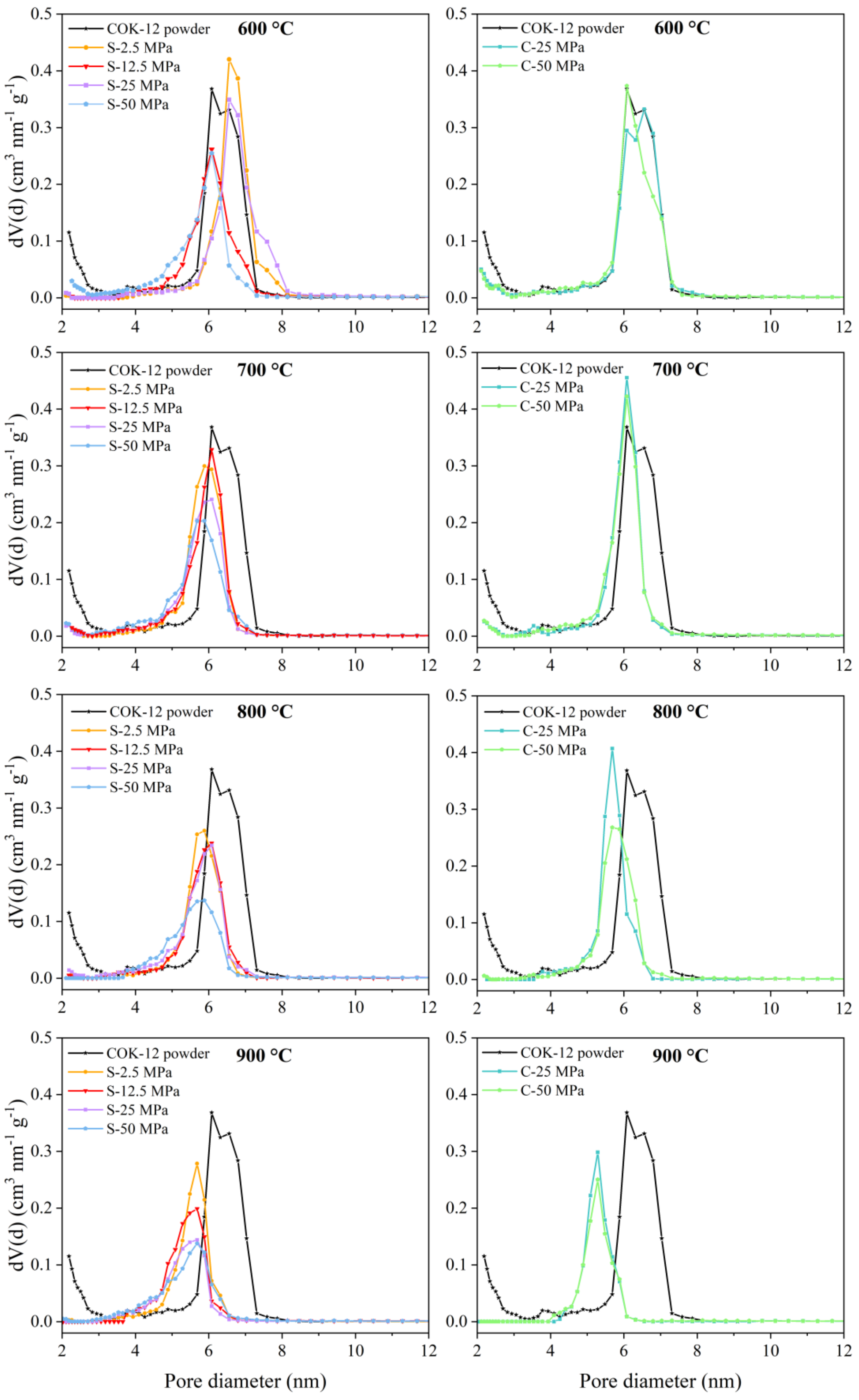

Figure S10. Nitrogen sorption pore size distributions (NLDFT, adsorption branch) of COK-12 powder and spark plasma sintered (SPS, S-sintered, left) and conventionally (C-sintered, right) COK-12 monoliths processed at pressures of 2.5–50 MPa and temperatures of 600–900 °C. No further peaks were found above 12 nm up to the limit of 80 nm.

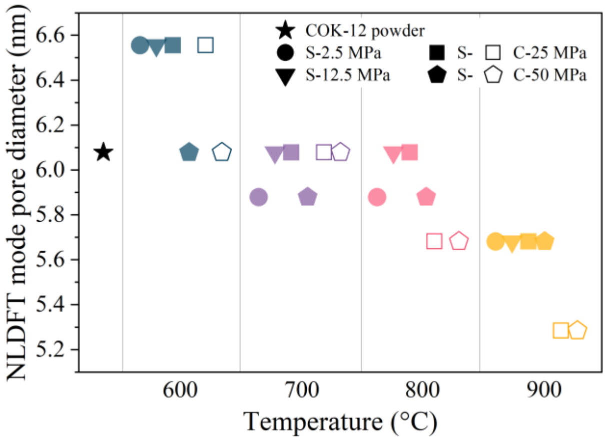

Figure S11. Mode pore diameter (NLDFT, adsorption branch) in dependence of the sintering temperature and pressure for spark plasma sintered (SPS, S-sintered) and conventionally (C-sintered) samples produced with a dwell time of 1 min and 12 h, respectively, and COK-12 powder.

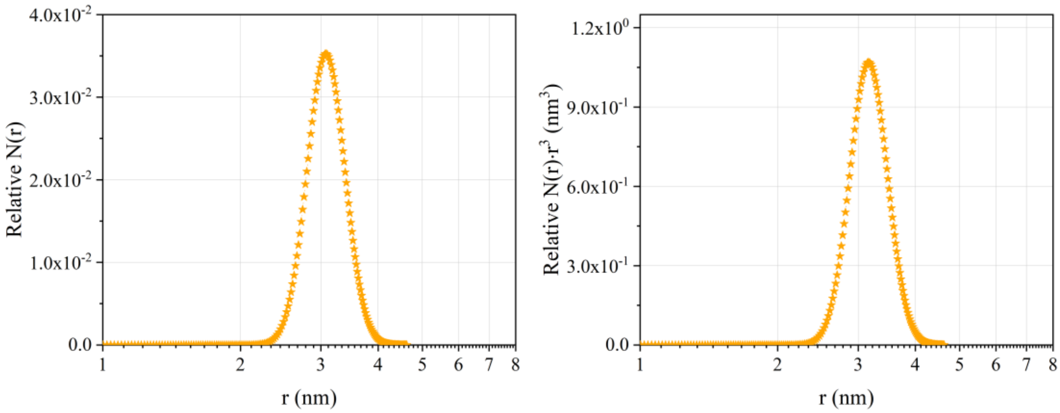

Figure S12. Number-weighted (left) and volume-weighted (right) pore size distribution of the mesopore radius of the loosely compacted COK-12 powder obtained by SAXS fitting.

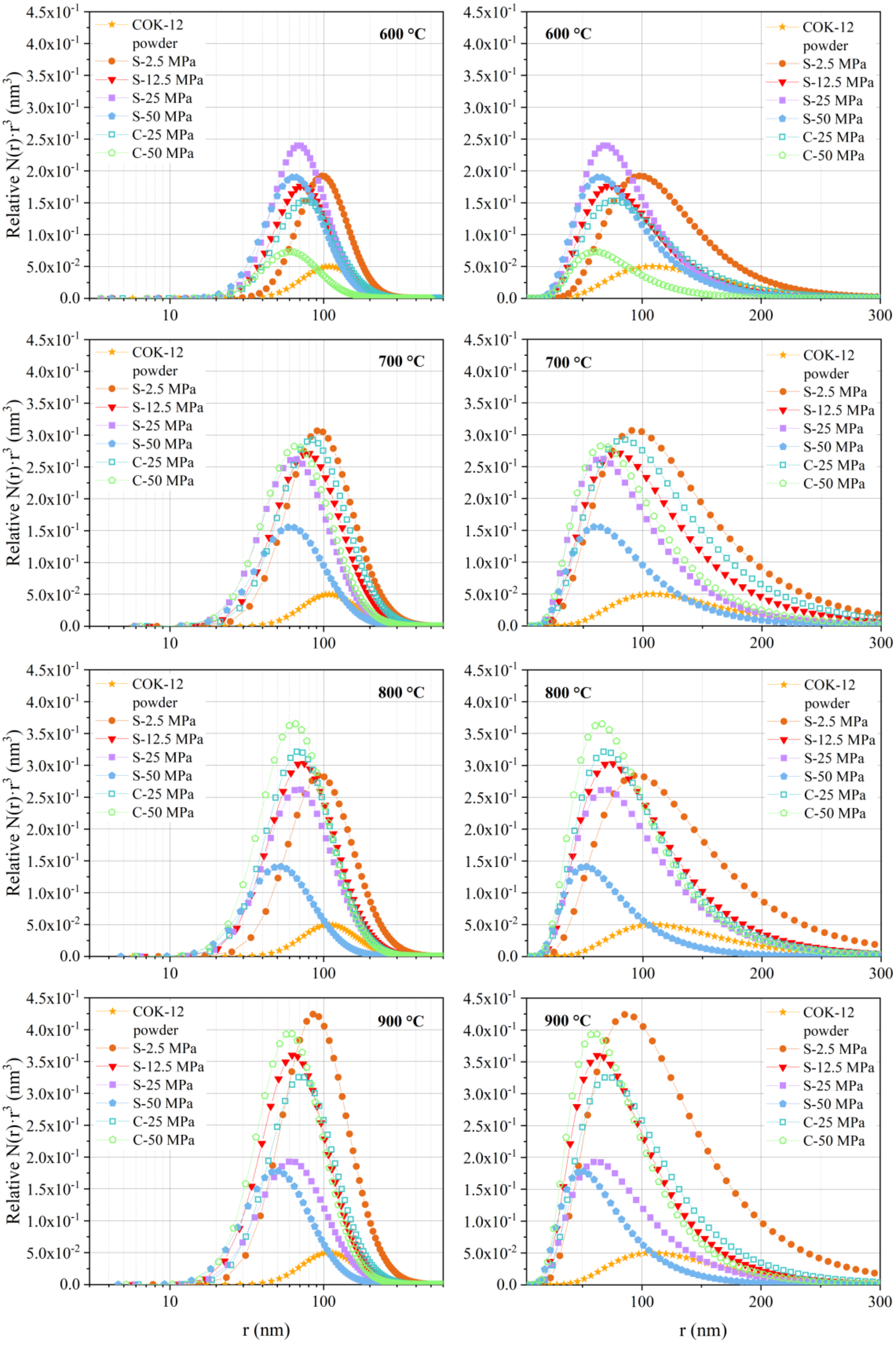

Figure S13. Volume-weighted pore size distributions of the macropore radii of the loosely compacted COK-12 powder and spark plasma sintered (SPS, S-sintered) and conventionally (C-sintered, right) monoliths obtained by SAXS fitting on a log scale (left) and linear scale (right).

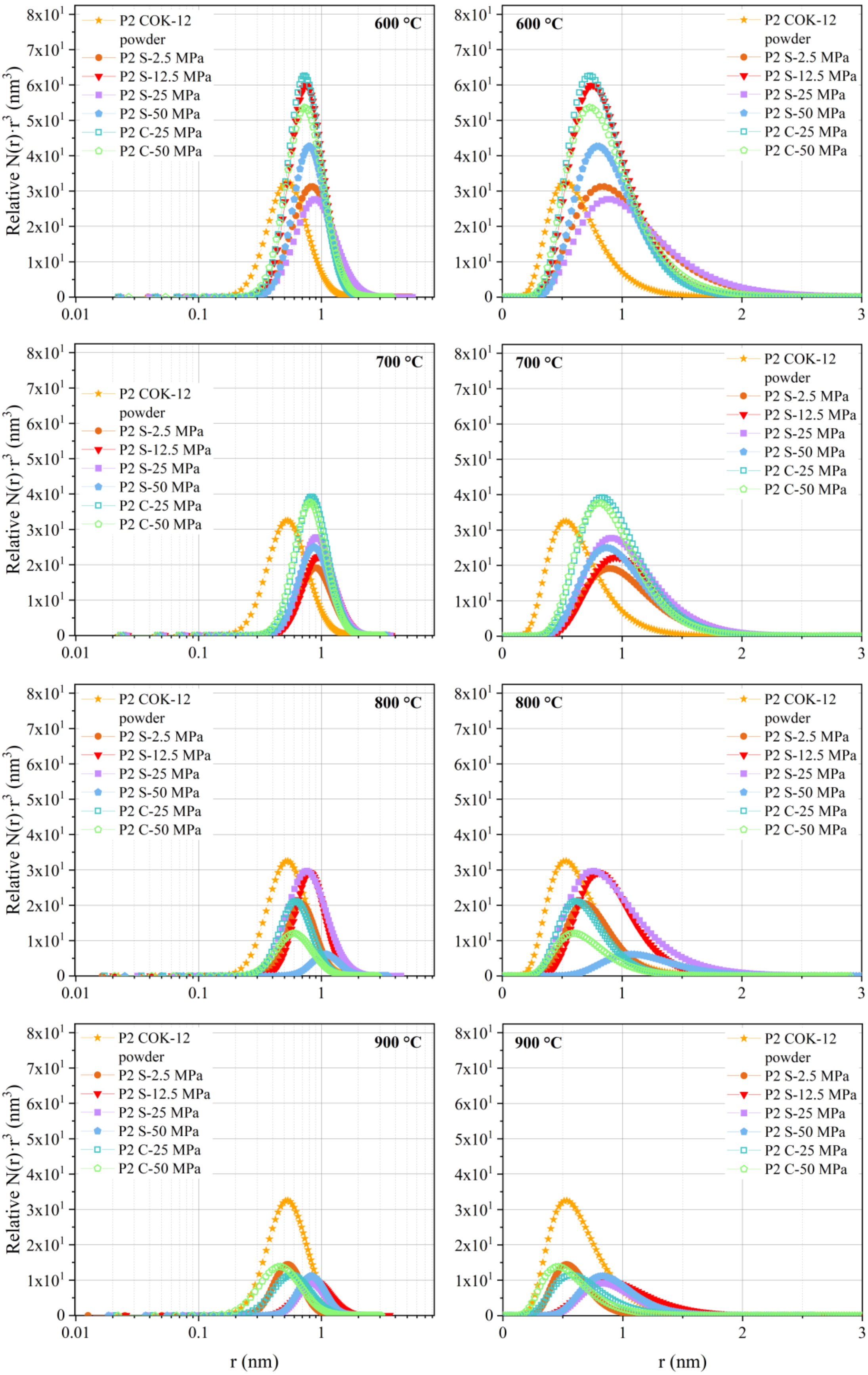

Figure S14. Volume-weighted pore size distributions of the P2 micropore radii of the COK-12 powder and and spark plasma sintered (SPS, S-sintered) and conventionally (C-sintered) monoliths obtained by SAXS fitting on a log scale (left) and linear scale (right).

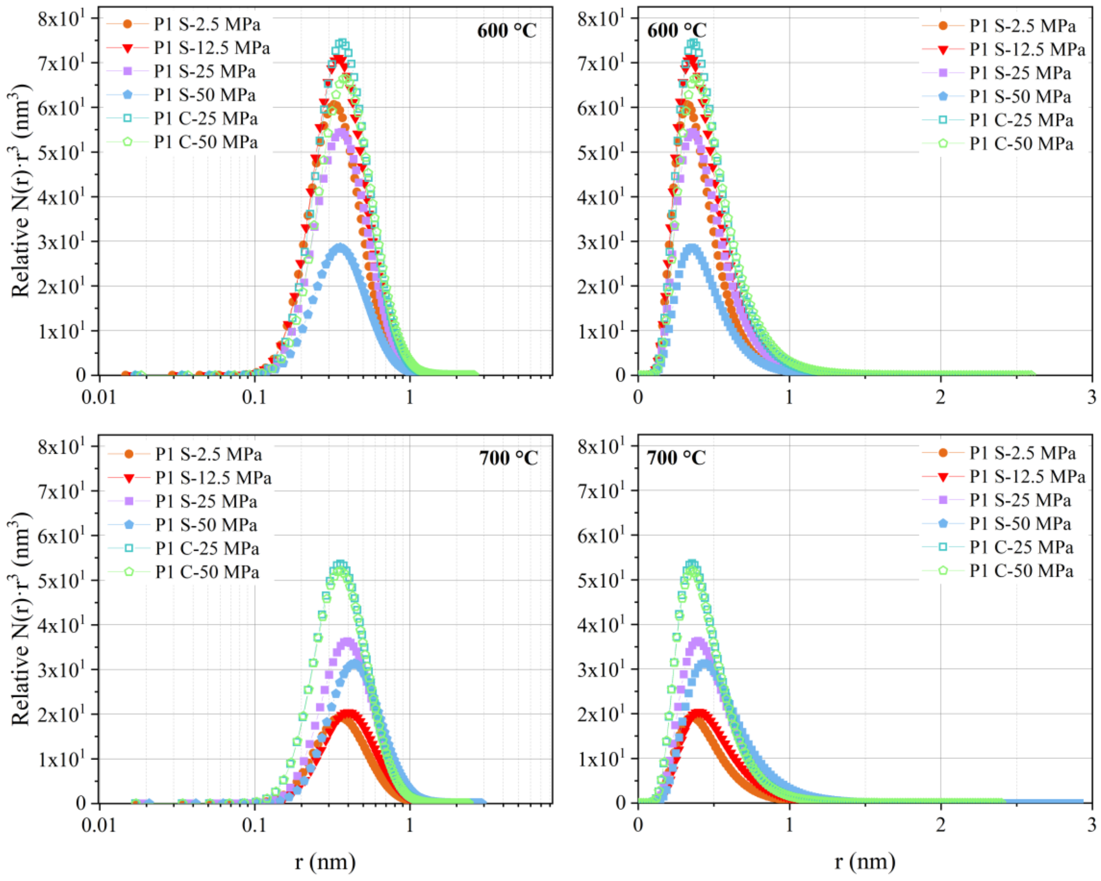

Figure S15. Volume-weighted pore size distributions of the P1 micropore radii of spark plasma sintered (SPS, S-sintered) and conventionally (C-sintered) monoliths at 600 and 700 °C obtained by SAXS fitting on a log scale (left) and linear scale (right).

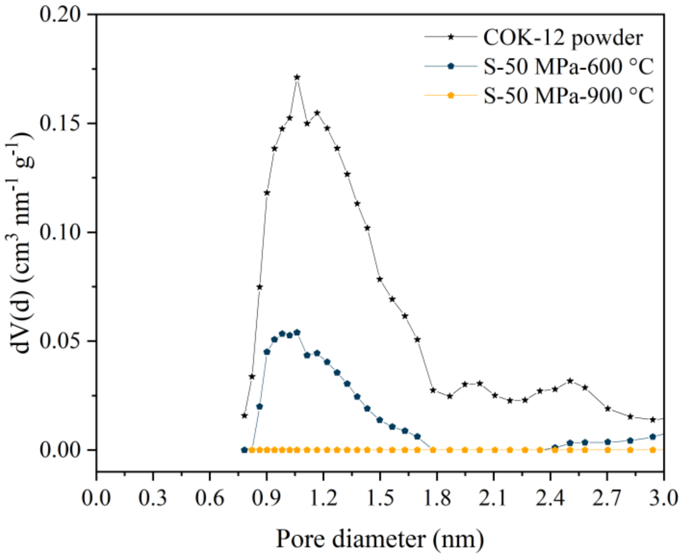

Figure S16. Nitrogen sorption micropore size distributions (NLDFT, adsorption branch) of COK-12 powder and spark plasma sintered (SPS, S-sintered) COK-12 monoliths processed at 50 MPa and temperatures of 600 and 900 °C, respectively. For the COK-12 powder, the micropores of a similar size are observed as compared to the SAXS fitting (Figure S14, Table S1). For the monolith processed at 50 MPa and 600 °C, a strongly assymmetric micropore size distribution can be observed. As the

SAXS fitting does only allow for a symmetric log normal distribution, two separate micropore entities were obtained from the SAXS fitting. When looking at the monolith processed at 50 MPa and 900 °C, no micropores can be observed from the nitrogen sorption measurement, yet a micropore contribution was required to fit the SAXS data. Such a contribution is required in SAXS and may not be observable in the sorption measurements possibly due to the significantly lower pore volume, namely about four times lower for S-50MPa-900°C in comparison to S-50MPa-600°C, compare Figure S14. Furthermore, these micropores might not be accessible, hence will be observable in the SAXS, due to the contrast variations, but not in the nitrogen sorption measurements.

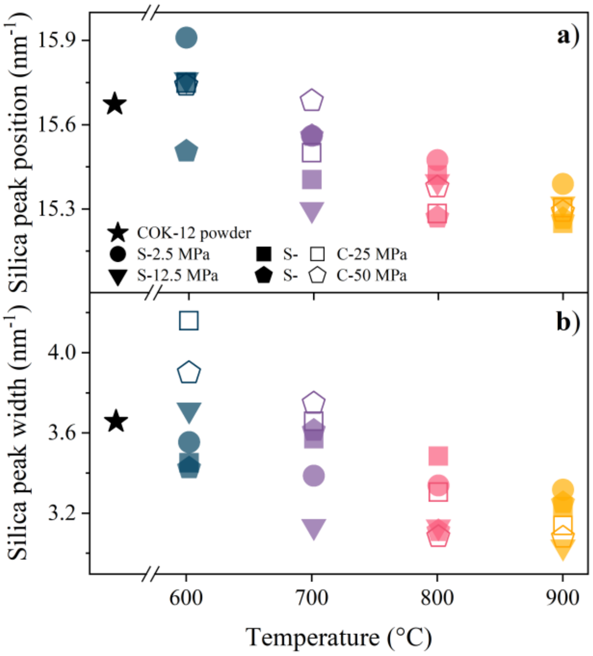

Figure S17. Silica a) peak positions and b) widths (half-width at half-maximum, HWHM) in reciprocal and real space for COK-12 powder and spark plasma sintered (SPS, S-sintered) and conventionally (C-sintered, right) monoliths as obtained from SAXS fitting.

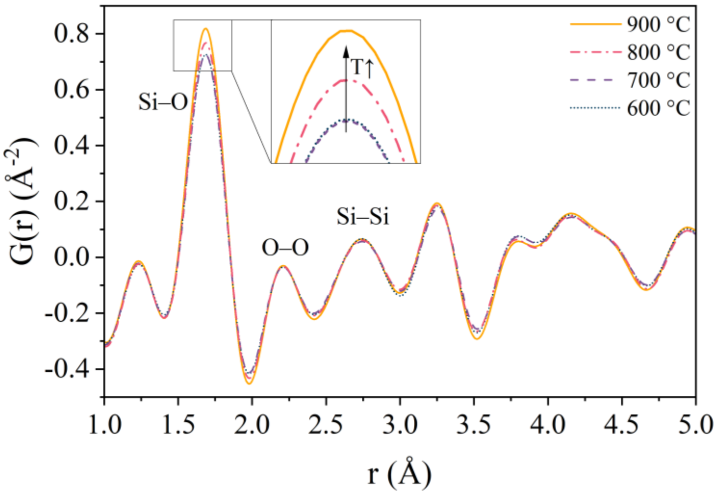

Figure S18. Pair distribution functions (PDFs) of the COK-12 monoliths produced by spark plasma sintering (SPS, S-sintering) at a pressure of 25 MPa and temperatures from 600 to 900 °C.

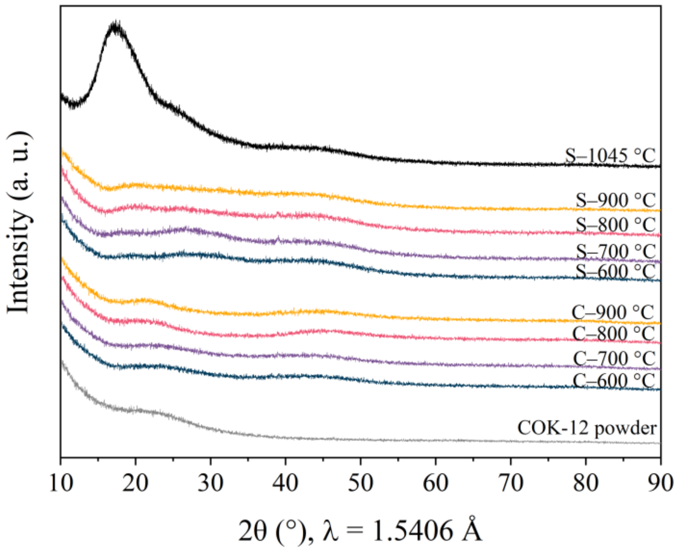

Figure S19. X-ray diffraction (XRD) pattern of COK-12 powder and spark plasma sintered (SPS, S-) and conventionally (C-) sintered samples produced at different sintering temperatures, showing that all samples remain amorphous up to 900 °C and a semi-crystalline behavior at 1045 °C. Pressure was 50 MPa for all samples. Dwell time was 1 min (45 min for 1045 °C, dense) and 12 h for the samples S- and C-sintered samples, respectively. Heating rate was 100 K min^{-1} (25 K min^{-1} for 1045 °C, dense) and 5 K min^{-1} for S- and C-sintered samples, respectively.

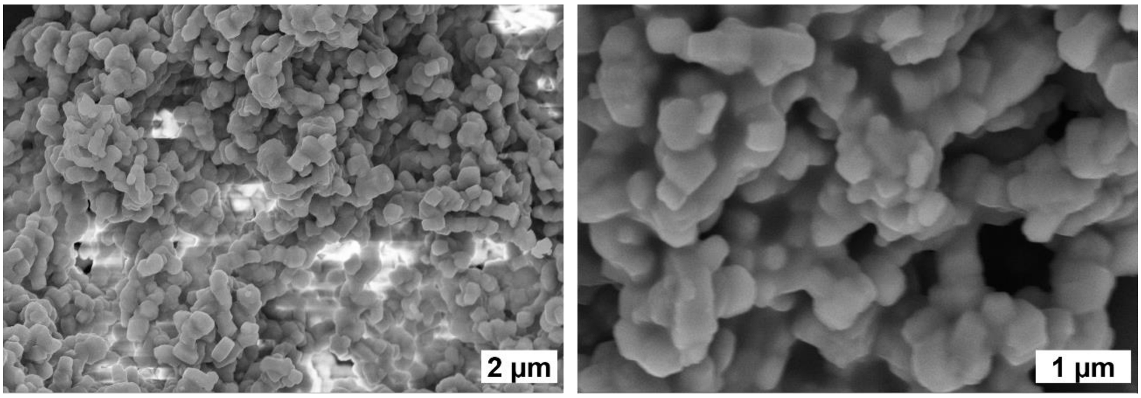

Figure S20. Scanning electron microscopy (SEM) images of the powder COK-12.

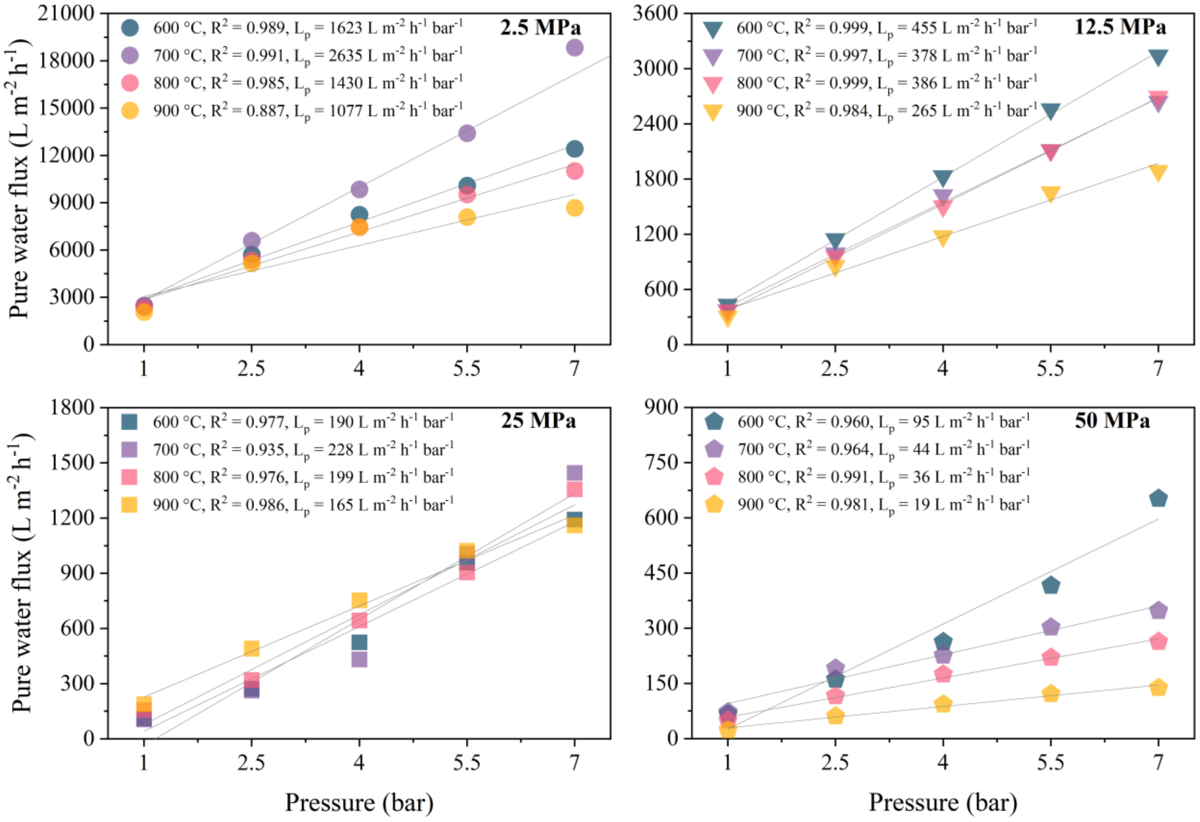

Figure S21. Pure water flux in dependence of the applied transmembrane pressure for spark plasma sintered (SPS, S-sintered) COK-12 monoliths produced at temperatures of 600–900 °C and pressures of 2.5–50 MPa and their corresponding linear fits and permeances. Please note the different ordinate scales.

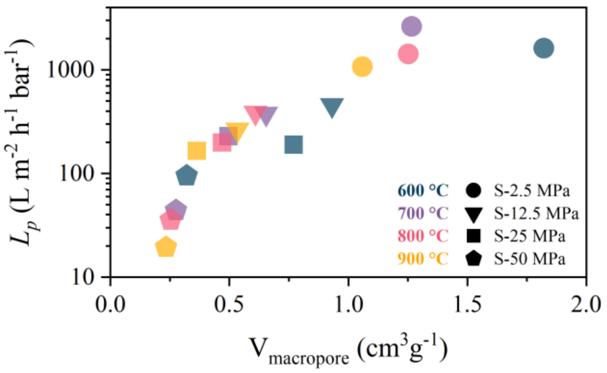

Figure S22. Relation between macropore volume and water permeance for spark plasma sintered (SPS, S-sintered) COK-12 monoliths. The macropore volume was estimated as $V_{macropore} = V_{total} - V_{N2}$ with $V_{total} = \varepsilon/100\rho_b$, compare **Table S1**.

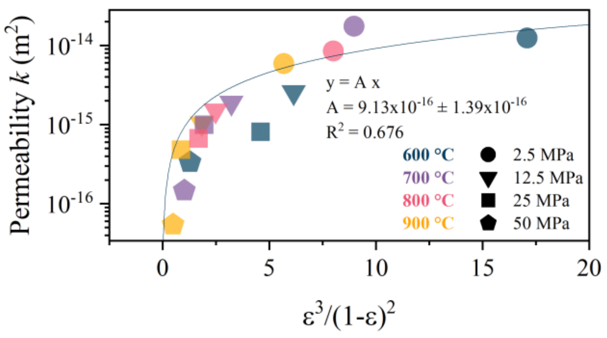

Figure S23. Fitting of modified Kozeny-Carman equation on complete spark plasma sintered (SPS, S-sintered) COK-12 monoliths dataset.

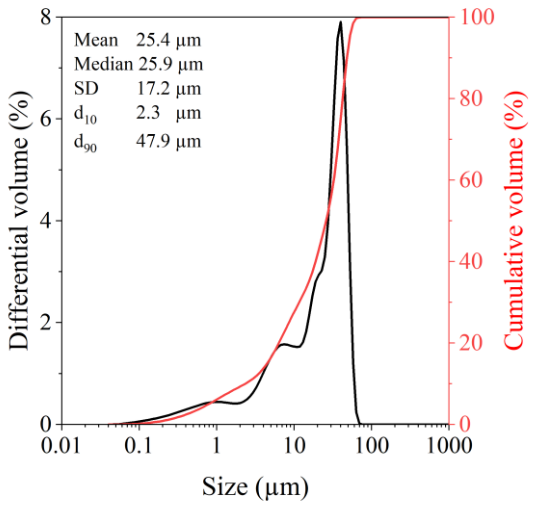

Figure S24. Oil droplet size distribution from the laser particle size analysis and corresponding characteristic values of the surfactant-stabilized oil in water emulsion used for the filtration experiment.

Table S1. Summary of the properties of COK-12 powder and monoliths produced by SPS (S) and conventional sintering (C).

M	P	T	S_{BET}	D_{DFT}	V_{N2}	$\boldsymbol{\varepsilon}$	ρ_b	ρ_a	σ	HV 9.807 N	D_{macro}	D_{meso}	D_{P2}	D_{P1}	a	w	w_A	L_p	k
	MPa	°C	$m^2 g^{-1}$	nm	$cm^3 g^{-1}$	%	$g cm^{-3}$	$g cm^{-3}$	MPa	MPa	nm	nm			nm	nm	nm^2	$L m^{-2} h^{-1} bar^{-1}$	m^2
COK-12 powder			645	6.1	0.61	N/A	N/A	N/A	N/A	N/A	256	6.2	1.21	N/A	10.4	4.2	31.8	N/A	N/A
S	2.5	600	300	6.6	0.42	82.0 ± 0.8	0.37 ± 0.01	2.04 ± 0.03	1.2 ± 0.3	7 ± 1	227	6.2	1.94	0.75	10.1	3.9	29.4	1623 ± 98	$1.25x10^{-14}$
S	12.5	600	428	6.6	0.47	74.2 ± 0.4	0.53 ± 0	2.05 ± 0.02	4.2 ± 0.3	35 ± 3	173	6.2	1.66	0.80	10.2	4.1	30.5	455 ± 9	$2.57x10^{-15}$
S	25	600	307	6.6	0.44	71.7 ± 0.8	0.60 ± 0.02	2.06 ± 0.02	8.0 ± 1.4	62 ± 3	162	6.1	2.05	0.83	10.3	4.2	31.1	190 ± 17	$8.12x10^{-16}$
S	50	600	330	6.1	0.37	59.5 ± 0.5	0.86 ± 0.01	2.10 ± 0.01	25.5 ± 0.8	251 ± 11	156	5.8	1.75	0.82	10.0	4.2	29.9	95 ± 11	$3.37x10^{-16}$
S	2.5	700	307	5.9	0.37	77.3 ± 0.4	0.47 ± 0.01	2.08 ± 0.01	2.9 ± 0.2	32 ± 5	236	5.5	1.96	0.83	9.5	4.0	27.5	2634 ±143	$1.76x10^{-14}$
S	12.5	700	308	6.1	0.38	68.4 ± 0.6	0.67 ± 0.01	2.11 ± 0.01	11.2 ± 1.6	84 ± 5	200	5.8	2.07	0.93	9.7	3.9	27.8	378 ± 12	$1.90x10^{-15}$
S	25	700	297	6.1	0.32	63.6 ± 0.8	0.78 ± 0.02	2.13 ± 0.01	15.9 ± 1.9	169 ± 10	165	5.8	2.00	0.91	9.9	4.1	29.0	169 ± 8	$9.99x10^{-16}$
S	50	700	315	5.9	0.35	57.1 ± 0.5	0.91 ± 0.01	2.14 ± 0.01	29.0 ± 3.4	337 ± 14	156	5.9	1.90	1.02	9.9	4.0	28.7	44 ± 5	$1.47x10^{-16}$
S	2.5	800	255	5.9	0.30	76.4 ± 0.8	0.49 ± 0.02	2.10 ± 0.01	3.3 ± 0.4	25 ± 2	240	5.7	1.47	N/A	9.4	3.7	25.4	1430 ± 102	$8.55x10^{-15}$
S	12.5	800	242	6.1	0.31	66.0 ± 0.9	0.72 ± 0.02	2.12 ± 0.01	12.8 ± 1.5	96 ± 4	184	5.7	1.74	N/A	9.7	4.0	27.8	386 ± 3	$1.51x10^{-15}$
S	25	800	273	6.1	0.30	62.2 ± 0.9	0.81 ± 0.01	2.13 ± 0.01	19.4 ± 0.3	195 ± 9	175	5.6	1.73	N/A	9.7	4.1	28.8	199 ± 18	$6.69x10^{-16}$
S	50	800	185	5.9	0.24	51.2 ± 2.2	1.0 ± 0.05	2.13 ± 0.01	32.5 ± 3.3	736 ± 50	131	5.7	2.31	N/A	9.8	4.1	28.9	36 ± 2	$1.04x10^{-16}$
S	2.5	900	226	5.7	0.27	73.5 ± 1.6	0.56 ± 0.04	2.11 ± 0.02	3.4 ± 0.6	37 ± 6	221	5.6	1.14	N/A	9.3	3.8	25.7	1077 ± 222	$5.94x10^{-15}$
S	12.5	900	217	5.7	0.27	63.1 ± 1.0	0.79 ± 0.02	2.14 ± 0.01	14.3 ± 1.5	157 ± 6	162	5.3	1.90	N/A	9.3	4.0	26.5	265 ± 19	$1.03x10^{-15}$
S	25	900	182	5.7	0.22	55.5 ± 2.5	0.96 ± 0.06	2.16 ± 0.01	25.0 ± 2.9	266 ± 11	159	5.5	1.46	N/A	9.5	4.0	27.0	165 ± 11	$4.82x10^{-16}$
S	50	900	200	5.7	0.23	49.6 ± 1.8	1.08 ± 0.03	2.16 ± 0	48.6 ± 1.8	724 ± 48	126	5.7	1.77	N/A	9.7	4.0	28.2	19 ± 2	$5.47x10^{-17}$
S	50	1045	N/A	N/A	N/A	0	2.20 ± 0.03	2.20 ± 0.03	124.3 ± 18.4	5360 ± 331	N/A	N/A	N/A	N/A	N/A	N/A	N/A	N/A	N/A

C	25	600	451	6.6	0.50	77.3 ± 0.4	0.47 ± 0.01	2.07 ± 0	1.9 ± 0.1	15 ± 1	185	6.1	1.61	0.85	10.1	4.1	29.9	N/A	N/A
C	50	600	434	6.1	0.48	72.9 ± 0.1	0.56 ± 0.00	2.07 ± 0.01	4.2 ± 0.4	35 ± 2	144	6.1	1.65	0.90	10.1	4.0	29.6	N/A	N/A
C	25	700	352	6.1	0.40	75.4 ± 0.4	0.52 ± 0.01	2.10 ± 0.02	1.9 ± 0.5	23 ± 2	215	6.1	1.82	0.83	9.9	3.7	27.2	N/A	N/A
C	50	700	350	6.1	0.41	71.5 ± 0.4	0.60 ± 0.01	2.10 ± 0	4.2 ± 0.5	49 ± 4	171	6.0	1.77	0.83	9.8	3.7	27.1	N/A	N/A
C	25	800	262	5.7	0.34	73.0 ± 0.3	0.57 ± 0.01	2.11 ± 0	3.8 ± 0.5	33 ± 1	175	5.6	1.35	N/A	9.4	3.9	26.3	N/A	N/A
C	50	800	260	5.7	0.32	68.6 ± 0.3	0.67 ± 0.00	2.14 ± 0.01	7.5 ± 1.0	77 ± 3	163	5.7	1.34	N/A	9.5	3.8	26.2	N/A	N/A
C	25	900	193	5.3	0.24	70.2 ± 0.4	0.64 ± 0.01	2.14 ± 0.01	4.0 ± 0.8	67 ± 5	181	5.3	1.29	N/A	9.1	3.9	25.2	N/A	N/A
C	50	900	181	5.3	0.22	65.5 ± 0.8	0.74 ± 0.01	2.15 ± 0.01	7.8 ± 0.2	97 ± 6	151	5.2	1.07	N/A	9.0	3.8	24.7	N/A	N/A

M	Monolith processing: SPS (S), conventional sintering (C)	HV 9.807 N	Hardness determined by Vickers indentation
P	Processing pressure	D_{macro}	Median macropore diameter determined by SAXS
T	Processing temperature	D_{meso}	Mesopore diameter determined by SAXS
S_{BET}	Specific surface area estimated by BET method	D_{P2}	Median larger micropore diameter determined by SAXS
D_{N2}	Pore diameter estimated by NLDFT on N_2 adsorption branch	D_{P1}	Median smaller micropore diameter determined by SAXS
V_{N2}	Pore volume estimated by NLDFT	a	Lattice parameter determined by SAXS
ε	Apparent porosity determined by Archimedes method	w	Wall thickness acc. to Eq. (5)
ρ_b	Bulk density determined by Archimedes method	w_A	Wall area calculated acc. to Eq. (6)
ρ_a	Apparent density determined by Archimedes method	L_p	Pure water permeance
σ	Biaxial strength determined by B3B method	k	Pure water permeability
N/A	not available		

Table S2. Fitting parameters for the biaxial strength, σ_0=124.3 MPa.

Model	Processing	R^2	Fitting parameter
Bal'shin	S	0.829	2.12
	C	0.928	2.75
Ryshkewitch	S	0.504	3.79
	C	0.535	5.12
Hashin	S	0.630	228.46
	C	0.521	642.34
Percolation	S	0.865	1.91
	C	0.964	2.45

Table S3. Fitting parameters for the Vickers hardness, HV_0 =5360 MPa.

Model	Processing	R^2	Fitting parameter
Bal'shin	S	0.947	3.59
	C	0.928	3.84
Ryshkewitch	S	0.560	6.59
	C	0.573	6.98
Hashin	S	0.399	43.38
	C	0.430	59.84
Percolation	S	0.962	3.24
	C	0.956	3.44

Table S4. List of studentized residuals for the fitting shown in **Figure S23**.

Independent Variable	Studentized residual
6.141	-1.052
4.590	-1.151
1.283	-0.279
5.669	0.261
1.835	-0.215
0.859	-0.101
0.486	-0.130
17.077	-1.490
8.963	**3.391**
3.219	-0.352
1.949	-0.262
1.011	-0.259
7.992	0.444
2.482	-0.255
1.688	-0.292

References

1 I. Breßler, J. Kohlbrecher and A. F. Thünemann, SASfit: a tool for small-angle scattering data analysis using a library of analytical expressions, *J. Appl. Crystallogr.*, 2015, **48**, 1587–1598.

2 K. Aratsu, R. Takeya, B. R. Pauw, M. J. Hollamby, Y. Kitamoto, N. Shimizu, H. Takagi, R. Haruki, S.-I. Adachi and S. Yagai, Supramolecular copolymerization driven by integrative self-sorting of hydrogen-bonded rosettes, *Nat. Commun.*, 2020, **11**, 1623.

3 H. Omar, G. J. Smales, S. Henning, Z. Li, D.-Y. Wang, A. Schönhals and P. Szymoniak, Calorimetric and Dielectric Investigations of Epoxy-Based Nanocomposites with Halloysite Nanotubes as Nanofillers, *Polymers*, 2021, **13**. DOI: 10.3390/polym13101634.

4 OpenSCAD 3D, http://openscad.org/.

5 SPONGE, https://github.com/BAMresearch/SPONGE.
